# Predicting the Minimal Translation Apparatus: Lessons from the Reductive Evolution of *Mollicutes*

**DOI:** 10.1371/journal.pgen.1004363

**Published:** 2014-05-08

**Authors:** Henri Grosjean, Marc Breton, Pascal Sirand-Pugnet, Florence Tardy, François Thiaucourt, Christine Citti, Aurélien Barré, Satoko Yoshizawa, Dominique Fourmy, Valérie de Crécy-Lagard, Alain Blanchard

**Affiliations:** 1 Centre de Génétique Moléculaire, UPR 3404, CNRS, Université Paris-Sud, FRC 3115, Gif-sur-Yvette, France; 2 INRA, UMR 1332 de Biologie du Fruit et Pathologie, Villenave d'Ornon, France; 3 Univ. Bordeaux, UMR 1332 de Biologie du Fruit et Pathologie, Villenave d'Ornon, France; 4 Anses, Laboratoire de Lyon, UMR Mycoplasmoses des Ruminants, Lyon, France; 5 Université de Lyon, VetAgro Sup, UMR Mycoplasmoses des Ruminants, Marcy L'Etoile, France; 6 Centre International de Recherche en Agronomie pour le Développement, UMR CMAEE, Montpellier, France; 7 INRA, UMR1225, Ecole Nationale Vétérinaire de Toulouse, Toulouse, France; 8 Université de Toulouse, INP-ENVT, UMR1225, Ecole Nationale Vétérinaire de Toulouse, Toulouse, France; 9 Univ. Bordeaux, Centre de bioinformatique et de génomique fonctionnelle, CBiB, Bordeaux, France; 10 Department of Microbiology and Cell Science, University Florida, Gainesville, Florida, United States of America; National Institute of Genetics, Japan

## Abstract

*Mollicutes* is a class of parasitic bacteria that have evolved from a common *Firmicutes* ancestor mostly by massive genome reduction. With genomes under 1 Mbp in size, most *Mollicutes* species retain the capacity to replicate and grow autonomously. The major goal of this work was to identify the minimal set of proteins that can sustain ribosome biogenesis and translation of the genetic code in these bacteria. Using the experimentally validated genes from the model bacteria *Escherichia coli* and *Bacillus subtilis* as input, genes encoding proteins of the core translation machinery were predicted in 39 distinct *Mollicutes* species, 33 of which are culturable. The set of 260 input genes encodes proteins involved in ribosome biogenesis, tRNA maturation and aminoacylation, as well as proteins cofactors required for mRNA translation and RNA decay. A core set of 104 of these proteins is found in all species analyzed. Genes encoding proteins involved in post-translational modifications of ribosomal proteins and translation cofactors, post-transcriptional modifications of t+rRNA, in ribosome assembly and RNA degradation are the most frequently lost. As expected, genes coding for aminoacyl-tRNA synthetases, ribosomal proteins and initiation, elongation and termination factors are the most persistent (i.e. conserved in a majority of genomes). Enzymes introducing nucleotides modifications in the anticodon loop of tRNA, in helix 44 of 16S rRNA and in helices 69 and 80 of 23S rRNA, all essential for decoding and facilitating peptidyl transfer, are maintained in all species. Reconstruction of genome evolution in *Mollicutes* revealed that, beside many gene losses, occasional gains by horizontal gene transfer also occurred. This analysis not only showed that slightly different solutions for preserving a functional, albeit minimal, protein synthetizing machinery have emerged in these successive rounds of reductive evolution but also has broad implications in guiding the reconstruction of a minimal cell by synthetic biology approaches.

## Author Summary

In all cells, proteins are synthesized from the message encoded by mRNA using complex machineries involving many proteins and RNAs. In this process, named translation, the ribosome plays a central role. The elements involved in both ribosome biogenesis and its function are extremely conserved in all organisms from the simplest bacteria to mammalian cells. Most of the 260 known proteins involved in translation have been identified and studied in the bacteria *Escherichia coli* and *Bacillus subtilis*, two common cellular models in biology. However, comparative genomics has shown that the translation protein set can be much smaller. This is true for bacteria belonging to the class *Mollicutes* that are characterized by reduced genomes and hence considered as models for minimal cells. Using homology inference approach and expert analyses, we identified the translation apparatus proteins for 39 of these organisms. Although striking variations were found from one group of species to another, some *Mollicutes* species require half as many proteins as *E. coli* or *B. subtilis*. This analysis allowed us to determine a set of proteins necessary for translation in *Mollicutes* and define the translation apparatus that would be required in a cellular chassis mimicking a minimal bacterial cell.

## Introduction

*Mollicutes* constitute a monophyletic class that share a common ancestor with Gram-positive bacteria of low G+C content or *Firmicutes* but have adopted a parasitic life style (**Figure S1**) [1]. During their coevolution with their eukaryotic hosts, mollicutes progressively lost the genes coding for cell-wall synthesis enzymes and for enzymes involved in the synthesis of small metabolites, such as amino acids, nucleotides and lipids that were available in the host. As a result, mollicute genomes are much smaller (580–1,840 Kbp; eg: about 482–2,050 CoDing Sequences or CDSs, **Table S1**) than those of model bacteria such as *Escherichia coli* or *Bacillus subtilis* (4,639–4,215 Kbp; eg: 4,320–4,176 CDSs respectively). These bacteria have nevertheless retained the full capacity to synthesize DNA, RNA and all the proteins required to sustain a parasitic life-style. In addition most of them are still able to grow in axenic conditions in rich media usually containing 20% serum (see [2] for review); only the hemoplasmas and the *Candidatus* phytoplasma species have yet to be cultured *in vitro. Mollicutes* are therefore considered as the smallest and simplest known bacteria capable of autonomous multiplication [3], [4]. ‘Simple’ does not mean ‘simplistic’. One should not underestimate the elaborate solutions that mollicutes have used to solve problems related to their peculiar macromolecular organization and cellular compactness (discussed in [3], [5], [6] and references therein). From an evolutionary point of view, mollicutes should be considered as some of the most evolved prokaryotes that still have retained ability to perform the complex reactions that encompass DNA, RNA and protein synthesis, with possibly new tricks and inventions to make the most of their limited genetic capacities [7], [8]. For these reasons, specific *Mollicutes* strains have been used as a test bench to improve our understanding of the basic principles of a cell and for reconstructing a microbe that would function with a synthetic minimal genome (see [3], [4], [9], [10], [11] for examples).

Identification of essential proteins is a long-standing problem that is directly linked to the concept of a minimal cell [12]. The approaches used in *Mollicutes* to identify the set of essential genes have been: i) comparative genomic analyses to create an overview of the protein content in model mycoplasmas (notably *Mycoplasma genitalium* and *Mycoplasma pneumoniae*) [5], [13], [14], [15], ii) identification of genes that cannot be individually inactivated [16], [17], [18], [19], iii) reconstruction of synthetic genomes and transplantation into a recipient cell [10]. Depending on the *Mollicutes* species considered and the method of analysis, the number of essential genes varies from 256 to 422. For *M. genitalium*, 256 were identified by *in silico* comparative genomics analysis [15] but over 382 were found by saturation transposon mutagenesis experiments [16], [19]. For *Mycoplasma pulmonis* and *Mycoplasma arthritidis*, saturation transposon mutagenesis identified 422 and 417 essential genes respectively [17], [20].

Messenger-RNA-dependent protein synthesis is one of the most complex cellular processes both in its biogenesis and its function. For a cell with a reduced genome such as *M. genitalium*, more than 25% of the genome encoding capacity is mobilized to build this complex machinery [2]. The bacterial ribosome is a giant multicomponent complex of several millions of daltons, composed of 3 RNA species (5S, 16S and 23S rRNA) and many structural proteins (60–70). Together with other RNAs (tRNAs, tmRNA and RNA-P) and a large repertoire of enzymes and protein factors, this protein synthesis machinery allows translation of mRNAs into polypeptides according to precise rules. Comparative analysis of bacterial genomes reveals that the majority of genes coding for the ribosomal proteins, aminoacyl-tRNA synthetases, translation factors and several ribosome biogenesis/maturation enzymes are universal [7], [21] and essential [22], [23], [24]. Genes coding for enzymes involved in rRNA and protein processing, RNA or protein modification, and ribosome maturation RNases appear less important, as deleting these does not lead to severe growth defects, and are the most easily lost genes during genomic erosion in *Mollicutes* species (see below).

As the number of sequenced *Mollicutes* genomes has significantly increased, most of the phylogenetic sub-groups of this class of bacteria are now covered allowing for the analysis of the erosion of translation from an evolutionary perspective. This analysis defined the minimal set of proteins needed to sustain protein synthesis in various mollicutes. A major goal of this work was to identify the minimal set of proteins that can sustain ribosome biogenesis and translation of the genetic code in *Mollicutes* that are model organisms of choice for synthetic biology. Also, by careful analysis of the evolutionary pattern of gene losses and a few cases of gene gain in different individual *Mollicutes* species, light was shed on the progressive adaptation of an ancestral and complex cellular proteome towards a simpler, yet functional alternative one.

## Results and Discussion

### Prediction of proteins involved in translation machinery

#### Selection of *Mollicutes* species

*Mollicutes* have been subdivided by phylogenetic analysis into 5 main sub-groups: Spiroplasma, Pneumoniae, Hominis, Anaeroplasma and Asteroleplasma [1]. The sub-group Asteroleplasma, which includes the single species *Asteroleplasma anaerobium*, is marginal, and mixed with other *Firmicutes* species questioning its membership to the *Mollicutes* class [25]. With the exception of asteroleplasmas, which could not be included in this study because of the lack of genome sequences, *Mollicutes* represent a monophyletic class of bacteria. The Anaeroplasma group is most commonly referred as the AAP sub-group as it includes the *Acholeplasma* and *Anaeroplasma* genera together with the *Candidatus* phytoplasma species.

A set of 39 genomes from distinct species that sample the diversity within *Mollicutes* were selected among the 60 sequenced genomes available at the time of this study. These include 9 species from the Spiroplasma sub-group, 16 from the Hominis sub-group, 10 from the Pneumoniae sub-group and 4 from the AAP sub-group. Among these 39 species, 27 have an animal host, including 7 a human host. Among the 5 species that are associated with plants, 4 are pathogens transmitted by sap-sucking insects. Culture as free living cells in axenic conditions has been achieved for 33 out of the 39 selected species: the uncultured ones are 3 hemoplasmas (*Mycoplasma haemofelis*, *Mycoplasma haemocanis* and *Mycoplasma suis*) and 3 *Candidatus* phytoplasma species (*Ca*. Phytoplasma mali, *Ca*. P. australiense and *Ca*. P. asteri) – they are boxed within a red dotted line in **Figure S1**. The 39 corresponding genomes have sizes ranging from 0.58 Mbp (482 predicted CDS) to 1.84 Mbp (2,050 predicted CDS) for *M. genitalium* and *Spiroplasma citri*, respectively (**Table S1**).

#### Selection of bacterial protein queries

**Figure 1:**
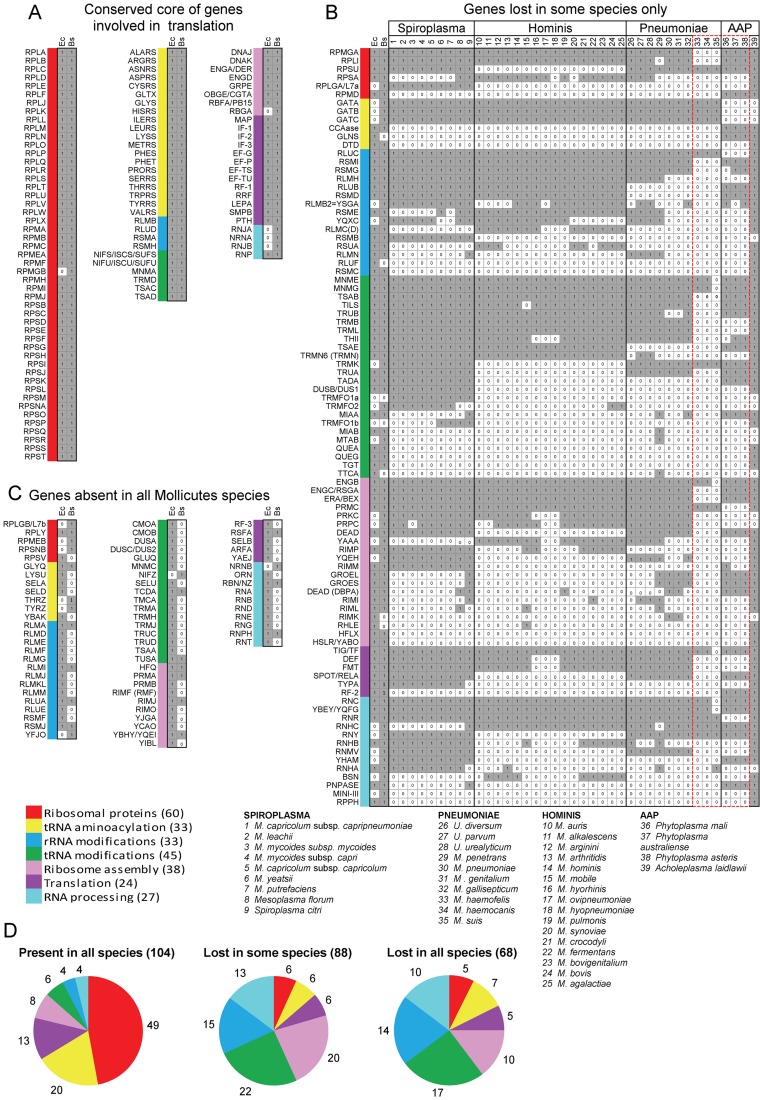
Genes coding for proteins implicated in translation in *Mollicutes.* Using queries from *E. coli* (*Ec*) and from *B. subtilis* (*Bs*), the presence of homologous proteins was searched in 39 *Mollicutes* genomes (see list of selected species below part B of the figure). This figure corresponds to the raw data given in **Table S3**. The results were grouped into three panels: conserved core of genes involved in translation (A), genes lost in some species only (B) and genes absent in all *Mollicutes* species (C). In panels A and C, only data concerning *Ec* and *Bs* are shown. In part B, the selected species clustered according to the 4 phylogenetic groups; Spiroplasma, Hominis, Pneumoniae and AAP [25]. The queries, of which names of corresponding acronyms are given in **Table S2**, are ordered from top to bottom, first according to the highest number of occurences and second according to the 7 protein categories following this sequence: ribosomal proteins, tRNA aminoacylation, rRNA modifications, tRNA modifications, ribosome assembly, translation and RNA processing. The different categories are color coded as shown in **Table 1** and below part C of the figure. The presence or absence of a given gene in a *Mollicutes* species is indicated by “1” in a grey background or by “0” in a white background, respectively. The 17 genes missing in some of the non-cultivated *Mollicutes* are indicated within a dashed-red box. The total number of genes in each category is indicated in panel D.

Our work deals exclusively with the mechanistic aspect of RNA-to-Proteins machinery and not with the transcription of DNA-to-RNA. We first had to define the set of protein queries. The Gram-negative bacterium *E. coli* is the organism for which almost all components of the translation machinery have been identified and experimentally characterized and this set was used as a starting point [26]. Since *Mollicutes* species are phylogenetically closer to Gram-positive *Firmicutes* than to Gram-negative *E. coli*, additional proteins from *B. subtilis* were also used [27]. Although *B. subtilis* homologs exist for most of the *E. coli* proteins involved in translation, there are a few *B. subtilis* translation proteins for which no homologs are found in the *E. coli* genome and vice-versa (**Table S2**). Altogether, we selected 260 protein queries, of which 228 are encoded by genes found in *E. coli*, 210 by genes found in *B. subtilis* and 179 are common between the two bacteria (**Table 1**). These proteins are involved in the biogenesis, maturation and proteosynthetic function of the ribosome and tRNAs. Not included were the proteins involved in RNA synthesis, in SRP/Sec-dependent membrane proteins translocation/secretion, in protein activation, in regulatory processes and in responses to stress or changes of environment and defense systems. The final 260 selected proteins were arbitrarily split into 7 categories according to their roles in the protein synthesis machinery (**Table 1**). For each of these 7 categories, a color code was used throughout the paper to facilitate understanding of the data (**Figure 1**).

**Table 1:** Genes coding for proteins implicated in the biosynthesis and functions of translation machinery in model bacteria.

Functional categories	Subcategories	Total of Escherichia coli queries	Total of Bacillus subtilis queries	Total of queries present in Ec and Bs	Total of queries not present in Ec	Total of queries not present in Bs	Total
**Ribosomal proteins**		55	59	54	5	1	**60**
	Large subunit proteins	33	37	33	4	0	
	Small subunit proteins	22	22	21	1	1	
**tRNA aminoacylation**		28	28	23	5	5	**33**
	AA-tRNA synthetases	23	24	21	2	2	
	Related Proteins	5	4	2	3	3	
**rRNA modifications**		30	23	20	3	10	**33**
	Specific for 23S rRNA	19	11	10	0	9	
	Specific for 16S rRNA	11	10	10	1	1	
	unknown target	0	2	0	2	0	
**tRNA modifications**		41	30	26	4	15	**45**
	Enzymes	35	24	21	3	14	
	Protein cofactors	8	6	5	1	1	
**Ribosome assembly**		33	30	26	5	7	**38**
	GTPases and factors	25	25	20	5	4	
	r-protein modifications	8	5	5	0	3	
**Translation**	GTPases and factors	24	20	20	0	4	**24**
**RNA processing**	RNases	17	20	10	10	7	**27**
	**TOTAL**	**228**	**210**	**179**	**32**	**49**	**260**

Ec: Escherichia coli.

Bs: Bacillus subtilis.

#### *Mollicutes* share a core of ubiquitous genes encoding proteins involved in translation

Inferring homology between each of the 260 protein queries and the predicted proteome of the 39 mollicutes was performed as described in Materials and Methods, using a combination of complementary approaches including sequence similarity searches, identification of conserved domains and phylogenetic analyses. The proteins involved in translation are known to be among the most conserved proteins in living organisms, which facilitated homolog predictions, especially in the monophyletic group of *Mollicutes*. The results of this data mining are summarized in the composite **Figure 1**.

In **Figure 1A** are listed the 104 genes that are present in the 39 genomes analyzed. The corresponding full names are given in **Table S3**. The presence of homologue genes in *E. coli* (Ec) and *B. subtilis* (Bs) are indicated in the small grey boxes adjacent to the acronyms. Only RpmGb, a duplicant of r-protein L31 of 50S subunit, is absent in *E. coli* (white small box). All these genes but three were shown to be essential in the model organisms (*E. coli*, *B. subtilis*) and/or in mycoplasmas (*M. genitalium*, *M. pulmonis* - **Figure S2, part A**). This core of ubiquitous genes represents 40% of the total queries (or 49% if only the genes present in *B. subtilis* are considered).

**Figure 1B** displays the 88 additional genes that have been lost (white small boxes) in at least one *Mollicutes* species. This data clearly shows that some genes are more persistent than others (i.e. the genes are conserved in a majority of genomes; [28]). Also, the non-culturable species (species 33 to 38, comprised in doted red box) have lost the most translation genes (vertical white small boxes), with several being lost only in *M. suis* (species 35) or in *M. suis* plus the two *M. haemofelis/canis* (species 33, 34). Out of these 17 persistent genes identified in non-culturable species, 14 are essential by gene deletion analysis in *M. genitalium* and/or *M. pneumoniae* (indicated in **Figure S2, B**, in orange background). Since non-cultivability is associated with the loss of genes that are required for growth in axenic conditions [29], this set of 17 genes should be considered as essential elements of a minimal translation machinery (discussed below). In all other cases, the individual genes are often absent in *Mollicutes* from different sub-groups. A few of these were found to be essential when tested individually in *M. genitalium* and/or *M. pulmonis* (**Figure S2, B**). All other genes are dispensible or can easily be lost because of the presence of paralogous or analogous genes with redundant or overlapping functions (discussed below). Most genes were lost early during *Mollicutes* evolution and subsequent genome downsizing. In a few cases, a gene present in a single or in a limited set of *Mollicutes* species but absent in *B. subtilis*, may correspond to a lateral gene transfer event (discussed below).

In **Figure 1C** are listed the 68 genes missing in all 39 mollicutes. Most are genes present in the Gram positive *B. subtilis* but absent in the Gram negative *E. coli*. Some of these could have emerged later during the evolution, after the separation of *Firmicutes* from other bacteria.

#### Some genes are more easily lost than others

As shown in **Figure 1D**, the genes that are the most easily lost in *Mollicutes* code for proteins involved in post-transcriptional modifications of t+rRNA (indicated in blue and green), in ribosome biogenesis and maturation – including post-translational modifications of ribosomal proteins (in pink), and ribonucleases involved in t+r+mRNA processing (in light blue). In contrast, genes coding for ribosomal proteins (in red), aminoacyl-tRNA synthetases (in yellow) and a few related proteins such as aspartyl/glutamyl-tRNA amidotransferases as well as a subset of translation factors are among the genes most resistant to loss (yellow and magenta in **Figure 1D**).

#### The minimal translation apparatus set of proteins depends on the *Mollicutes* sub-groups

**Figure 2:**
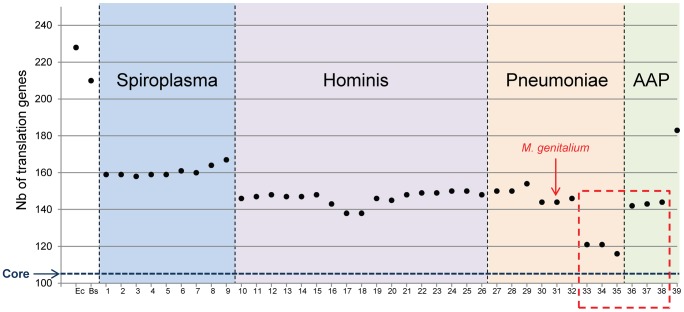
Total number of proteins involved in translation for each *Mollicutes* species. The number of proteins involved in translation for each *Mollicutes* species was tabulated in reference to the number found for the two model bacteria *E. coli* (Ec) and *B. subtilis* (Bs). The numbering of species is the same as in Figure 1. The data corresponding to non-cultivated *Mollicutes* are framed with a red dashed line as in Figure 1. The horizontal blue dashed line indicates 104, which correspond to the core of translation proteins shared by all *Mollicutes.*

The total number of proteins involved in translation for each *Mollicutes* species was then tabulated (**Figure 2**). It is clear that gene erosion is not uniform in each sub-group of the *Mollicutes* tree and that different sets of persistent genes exist in each *Mollicutes* sub-groups. In other words there are different ways to evolve towards a minimal and functionally coherent cell. The Spiroplasma sub-group retained the largest numbers of genes (from 158 to 167). At the other extreme, the species that shed the most genes lost are the three hemoplasmas (116, 121 and 121 genes). At variance, the three phytoplasmas, which share with the hemoplasmas the inability to grow in axenic conditions, have a larger set of genes (142, 143 and 144 genes), closer to that found in the other mollicutes. Among them, two different minimal sets are found in the Hominis group (138 genes for *Mycoplasma ovipneumoniae*, and for *Mycoplasma hyopneumoniae*) and in Pneumoniae group (144 genes for the closely related *M. genitalium* and *M. pneumoniae*). These data also indicate that there is no strict relationship between genome sizes, cell cultivability and the number of genes dedicated to translation (compare **Figure 2** with genome sizes indicated in **Table S1**). Indeed, the hemoplasma *M. haemofelis* genome (1.1 Mbp) is larger than the phytoplasma genomes, and yet has over 26 less translation genes (see above). Similarly, the genome of *M. ovipneumoniae*, is almost twice the size of *M. genitalium*, and yet this species has a smaller number of translation genes (138 *vs* 144). This lack of correlation is not unexpected because genome downsizing during the *Mollicutes* evolution can be followed (or paralleled) by an expansion phase resulting from duplications [30], [31] and/or from acquisitions by lateral transfer [32], [33], [34], [35].

### Scenario for genome erosion during *Mollicutes* evolution

#### Overview: loss and gain of genes

**Figure 3:**
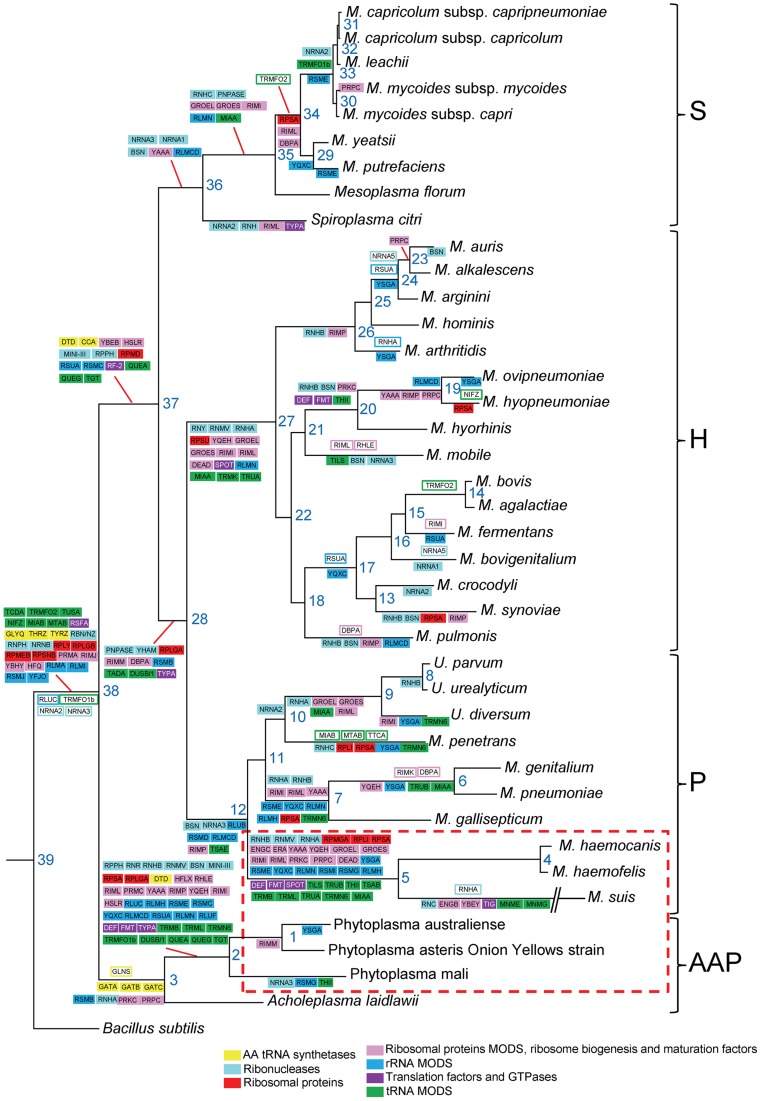
Reconstruction of the evolution of translation-related gene set in mollicutes. Ancestral gene content at each node of the phylogenetic tree was inferred using the posterior probabilities calculated from the birth-and death model implemented in the COUNT program. Genes gained and lost are framed and highlighted with colors corresponding to gene categories, respectively. Very similar results were obtained using Wagner parsimony method with a gain penalty of 4. The phylogenetic tree was inferred using the maximum likelihood method from the concatenated multiple alignments of 79 proteins encoded by genes present at one copy in each genome. The phylogenetic groups are indicated: S for Spiroplasma, H for Hominis, P for Pneumoniae and AAP. The non-cultivated *Mollicutes* are framed by a red dashed line.

Using our dataset of translation genes, we performed a reconstruction of gene gain and loss events in *Mollicutes* evolution. In this reconstruction, we hypothesized that the last ancestor common between the *Mollicutes* and *B. subtilis* was a virtual organism with 220 genes involved in translation (i.e. 208 *B. subtilis* query genes +12 genes found in *Mollicutes* but not in the modern *B. subtilis*). Ancestral gene content at each node of the phylogenetic tree was inferred using the posterior probabilities calculated from the birth-and death model implemented in the COUNT software package [36]. Taking into account that the genome downsizing was probably a major component in *Mollicutes* evolution, the scenario was built allowing no gene gain in *B. subtilis* (**Figure 3**). This evolutionary scenario is supported by the similar results obtained using the Wagner parsimony method with a high penalty for gene acquisition [37]; only 17 out of the 220 genes were found to have a different history in this reconstruction.

Using this method, we found 26 gene gain events involving 20 different genes. The acronyms of the corresponding genes are indicated in open boxes with lines corresponding to the color code as defined in **Table 1**. In contrast to these rare cases of gene gains, there were 255 gene losses with 3 major nodes totaling 99 loss events (39%): node 38 that represents the entrance in the *Mollicutes* class with 25 losses, node 2 that represents the separation between phytoplasmas and acholeplasmas with 38 gene losses and node 5 leading to the hemoplasmas with another 36 genes losses (**Figure 3**). Once again these results emphasize the particular status of the non-cultivated *Mollicutes* surviving with a minimal set of proteins (**Figure 3**). There are also other nodes showing major gene losses, including node 27 (15 losses) that corresponds to the separation of the Hominis group from the other mollicutes. This is quite remarkable because it involves a large cluster of species (16 altogether) that are characterized by a great diversity of animal hosts (**Table S1**). The events at node 38 are dependent of the arbitrary choice of *B. subtilis* as a model for last common ancestor for the *Mollicutes*

The various gains and losses of genes for each category of proteins considered (**Table 1**) in the 39 mollicutes are discussed below. The ones that have been lost in all *Mollicutes* (**Figure 1C**) are not systematically discussed.

#### Ribosomal proteins

The major function of ribosomal proteins (r-proteins) is stabilization of rRNA structure, although some of them are also involved in functional interactions with m+t+tmRNAs and translation factors. Of the 60 query genes coding for r-proteins present in ribosomes of *E. coli* and/or *B. subtilis*, 49 are present in the 39 mollicutes genomes examined (**Figure 1A** **, Table S3**). Five genes encoding r-proteins (S14b/RpsNb, the ribosomal associated protein SRA or S22/RpsV, L31b/RpmEb, L7b/RplGb and L25/RplY) are missing in all *Mollicutes* (**Figure 1C**). These could have been lost very early in the genomic erosion (node 38, **Figure 3**) or could have emerged later in *B. subtilis* lineage, after the separation of the *Mollicutes* lineage.

For the other r-proteins, the situation varies with the specific mollicute analyzed (**Figure 1B** and **Table S3**). In contrast with the S14, L31, L7a (RplGb) cases discussed above, where one of the two encoding paralogous genes is absent at the emergence of *Mollicutes* class, most species tend to retain the two genes encoding L33a (RpmGa) and L33b (RpmGb), L33a being lost only in the 3 hemoplasmas (node 5). Protein L9 (RplI) with two globular domains (one being exposed out of the 50S subunit) normally interacts with tRNA in the P site and limits mRNA slippage (frameshift) [38]. It is also lost in the 3 hemoplasmas (node 5) and in the single *Mycoplasma penetrans* (node 10).

S21 (RpsU) was lost only once at the root of the Hominis sub-group (node 27), whereas S1 (RpsA) was lost independently seven times (nodes 2, 5, 7, 10 13, 19 and 34), remaining in several species of the Hominis sub-group, and absent in most of the other mollicutes. S1 is important for translation initiation of Shine-Dalgano (SD)-containing mRNAs and becomes obsolete for reading leaderless-mRNAs [39]. Proteins S1 and S21, both playing a role in the initiation process, seem mutually exclusive. Finally, L30 (RpmD, lost at node 37) is found only in the AAP sub-group.

Of note, S1, S21, S22 (SRA), L7a, L25 and L30 are absent in many other bacterial species [21]. Together with L33a mentioned above, they are known to be responsible for cellular ribosome heterogeneity, probably generating specialized ribosomes in response to stress conditions and environmental changes [40]. These r-proteins could have arisen during evolution to fufil specific non-essential innovations [41], and hence could be easily lost during the reductive evolution of *Mollicutes* ribosomes. Moreover, systematic chromosomal deletion studies of bacterial r-protein genes showed that many of these (24/55 in *E. coli* and 22/57 in *B.subtilis*) were not essential (**Figure S2** and: [42], [43], [44] [45]).

#### Translation factors

In addition to the core ribosomal components, protein synthesis requires a series of translation factors. These factors ensure the speed and the fidelity of translation, as well as the functionality of the nascent polypeptide. Most of them are found in all *Mollicutes* illustrating again the conservation of the translation apparatus in the bacterial world. Translation factors present in all *Mollicutes* are the initiation factors IF1, IF2, IF3, the elongation factors EF-G, EF-P, EF-Ts and EF-Tu, the peptide chain release factor RF1, the recycling factor RRF, the back translocation elongation factor LepA (also designated EF4), the peptidyl hydrolase PTH, the methionine aminopeptidase (MAP) that releases non-formylated methionine from the N-terminal nascent peptide, and SmpB associated to tmRNA that rescues ribosomes stalled on truncated mRNAs. All of the above, except LepA, correspond to essential genes in bacteria including *M. genitalium* and *M. pneumoniae* (**Figure S2**). In *E. coli*, LepA becomes essential only under unfavourable growth conditions, such as low temperature or high ionic strength [46].

The ribosome-associated trigger factor TIG (also designated TF) is not essential in *E. coli* and is missing only in the non-culturable *M. suis*. In *M. genitalium*, TIG has two activities: the co-translational folding of nascent polypeptide and a peptidyl-prolyl isomerase activity [47], [48]. Together with DnaK/DnaJ/GrpE and GroEL/GroES, TIG belongs to the essential polypeptide chaperone networking system (see below and [49], [50].

A few translation factors are dispensable in several mollicutes. Methionyl-tRNA formyltransferase (FMT) that catalyzes the formylation of Met on initiator Met-tRNA^Meti^ and peptide deformylase (DEF) that subsequently removes the formyl group from the N-terminal methionine of translated peptides, are both absent in the six non-culturable hemoplasmas/phytoplasmas (nodes 2 and 5) and the three species of the Hominis subgroup, *Mycoplasma hyorhinis*, *M. ovipneumoniae* and *M. hyopneumoniae* (node 20 in **Figure 3**). The concomitant loss of both these proteins, while the methionine aminopeptidase (MAP) remains ubiquitous (**Figure 1B**), agrees with the observation that in *E. coli* the *def* gene could be inactivated only if the *fmt* gene was also inactivated [51].

Of the two *E. coli* ribosome-associated bi-functional stringent factors RelA and SpoT, only one (designated RelA/SpoT) is present in *Firmicutes* [52]. These bi-functional enzymes carry a GDP/GTP-dependent (p)ppGpp synthetase and a phosphohydrolase activity that regulate the concentration of the alarmone (p)ppGpp in response to various environmental stresses, such as temperature change, transition to the stationary phase, or limitation of essential metabolites. In *Mollicutes* RelA/SpoT is lost in all the Hominis species (node 27) and in the 3 hemoplasmas (node 5).

The GTPase TypA (or BipA), universally conserved in Bacteria, is another translation regulator that exhibits differential ribosome association in response to stress-related events [53]. Homolog of TypA is lost in all phytoplasmas, in all Hominis and Pneumoniae species (nodes 2 and 28), plus the single *S. citri*.

The release factor 2 (RF2), required for reading the UGA termination stop codon, is missing in all mollicutes but the three phytoplasmas and *A. laidlawii* (node 37, **Figure 3**). The UGA codon is decoded as Trp in all mollicutes lacking RF2 [54] by an extra tRNA^Trp^ harboring a U*CA anticodon [55]. In the case of *M. capricolum*, the wobble base (U*34) is post-transcriptionally modified to cmnm^5^U [56]. In agreement with RF2 being absent, two other proteins (ArfA and YaeJ) are also absent, another example of concerted elimination of proteins belonging to the same biochemical process. ArfA rescues stalled-ribosomes from mRNA by recruiting RF2 to release tRNA, and YaeJ hydrolyzes peptidyl-tRNA (without RF2) on stalled ribosomes. The use of UGA codon as a Trp codon in most *Mollicutes* species also agrees with the lack of co-translational incorporation system (SelA, SelB, SelC and SelD) [57] for selenocystein in mollicutes as it uses the same UGA codon.

#### Aminoacyl-tRNA synthetases and a few related proteins

All *Mollicutes* genomes analyzed encoded the complete set of aminoacyl-tRNA synthetases (aaRS) and protein cofactors required to charge all 20 canonical amino acids. They need only 19 classical aaRSs as the gene coding for glutaminyl-tRNA synthetases (GlnS), found in many other bacteria (including *E. coli*), is missing in most mollicutes [58] [59]. Like their *Firmicutes* progenitor, mollicutes encode a non-discriminating type of glutamyl-tRNA synthetase (GltX) that charges both tRNA^Glu^ and tRNA^Gln^ with Glu, and the heterotrimeric enzyme encompassing GatA, GatB and GatC (Gln-tRNA amidotransferase complex) that amidates Glu-tRNA^Gln^ to Gln-tRNA^Gln^ [60]. The loss of genes coding for the GatA/B/C enzymatic system and the gain of GlnS are concomitant and occurred at the root of the AAP sub-group (node 3 in **Figure 3**, see also in **Figure 1B**). This mutually exclusive process seemed to have occurred repeatedly in bacterial evolution [58].

Bacterial GlyRSs are of two types: a tetrameric form (α_2_β_2_) and a dimeric form (α_2_), the corresponding subunits being encoded by *glyS* (α subunit) and *glyQ* (β subunit) genes, respectively. *B. subtilis* str. 168 harbors a α_2_β_2_ type GlyRS, whereas other bacilli, such as *Bacillus anthracis* str. A2012 or *Bacillus thuringiensis* serovar konkukian str. 97-27, harbor an α_2_ type enzyme [61]. All mollicutes encode only GlyS and no GlyQ homologs (**Table S3**), suggesting that the homodimeric form of GlyRS was already present in the *Mollicutes* progenitor. PheRS is the only α_2_β_2_ heterodimeric aaRS found in all mollicutes, each subunit being encoded by the co-transcribed tandem *pheS* (for α subunit) and *pheT* (for β subunit) genes [62]. Interestingly in *M. pneumoniae*, the PheRS α_2_β_2_ was detected *in vivo* in a complex with four other synthetases (TyrS, MetG, ThrS, GltX) [11]. This multiprotein complex is reminiscent of the multi-synthetase complex found in Eukarya and Archaea, but elusive in Bacteria [63]. Lastly, a single gene is found for LysRS, TyrRS and ThrS, no duplicant for LysU, ThyZ and ThrR like in other bacteria.

Many aaRSs are prone to mistakes and mischarge structurally similar amino acids. To minimize mistranslation, these enzymes harbor an editing activity to hydrolyze mischarged tRNAs. In *Mollicutes*, several aaRS carry mutations or even deletions in their editing domains that increase mistranslation frequency. Such genetic variants have been identified in LeuRS, PheRS and ThrRS editing domain of several *Mycoplasma* species [64], [65], [66]. In addition, the *Mollicutes* ProRSs are of the eukaryotic/archaeal type that lack the cis-editing domain [67]. Finally, no homologs are found in any *Mollicutes* species of the stand-alone bacterial editing proteins like the YbaK, ProX or AlaX families that hydrolyze misacylated Cys-tRNA^Pro^, Ala-tRNA^Pro^, Ser-tRNA^Ala^ and Gly-tRNA^Ala^, respectively [68]. The systematic absence of aaRS editing functions in mollicutes suggested high misincorporation rate that were experimentally validated in a few cases [64], leading to a ‘statistical proteome’ that could be one of the reasons *Mollicutes* species are evolving faster than any other extant bacteria (discussed in: [69], [70], [71]). In *E. coli*, a D-aminoacyl-tRNA deacylase (dtd, *yihZ* gene in *E. coli*) allows recycling the D-containing misaminoacylated tRNA [72]. Orthologs of the *yihZ* gene occur in nearly all bacteria, including *B. subtilis (yrvI)* but in *Mollicutes* only *A. laidlawii* harbors a *yihZ* homolog.

Finally, because the terminal 3′-CCA sequence of mature tRNAs in all *Mollicutes* species are generally encoded in the genome [73], the tRNA nucleotidyl transferase (CCAase) became obsolete and the pre-tRNA processing machinery exactly trims the tRNA at the CCA end with one RNase only [74], while in other bacteria several accessory RNases are needed (see below). The loss of the encoded CCAase gene occurred very early in *Mollicutes* evolution (node 37, **Figure 3**). As a consequence, one can expect the absence of 3′-CCA end turnover and of repair of tRNAs lacking the terminal amino acceptor adenosine. The systematic presence of CCA sequence at the end of all tRNA primary transcripts, instead of longer 3′-tail as in majority of bacteria, exemplifies again the genome economy strategies of mollicutes.

#### Transfer RNA modification enzymes

tRNA precursors are subject to enzymatic post-transcriptional modifications at many positions of the base or ribose moieties. These modifications stabilize the tRNA tertiary structure, introduce recognition determinants and antideterminants towards RNA-interacting macromolecules and fine-tune the decoding process at the level of both efficiency and fidelity. Genes coding for almost all *E. coli* tRNA modification enzymes have been identified and experimentally verified, and most of them have homologs in *B. subtilis*. A few additional *B. subtilis* genes coding for enzymes that are absent in *E. coli* have also been characterized (**Table S3** and **Figure S3**).

Of the 45 query genes coding for tRNA modification enzymes only a handful of homologs are predicted to resist genomic erosion in *Mollicutes*. These encode the two proteins TsaC and TsaD of the multienzymatic complex involved in t^6^A formation composed of 4 subunits in *E. coli* (TsaB, TsaC, TsaD, TsaE) [75], the site-specific methyltransferase TrmD catalyzing formation of m^1^G, and thiouridine synthetase MnmA catalyzing the thiolation of wobble uridine (s^2^U). All these modifications are located in the anticodon loop (position 34 or 37) of a subset of tRNAs (**Figure S3**). Of the other proteins of the t^6^A synthesis machinery, TsaB is missing in the 3 hemoplasmas (node 5, **Figure 3**), while TsaE is missing in all species of the Pneumoniae subgroup (node 12, **Figure 3**). In these latter organisms the t^6^A machinery is reminiscient of the recently elucidated mitochondrial pathway also composed of only two proteins [76].

In *Mollicutes*, the sulfur relay system working in conjunction with MnmA has yet to be characterized. Of the complex sulfur relay encompassing at least 7 components (IscU/IscS/TusA/TusB/TusC/TusD/TusE) identified in *E. coli* but not in *B. subtilis* [77], only IscU and IscS are present in all mollicutes. The most parsimonious explanation would be that the cysteine desulfurase IscS/IscS/NifS and/or the alternative SufU/SufU/NifU present also in *B. subtilis* suffice to provide the sulfur moiety by direct transfer of the sulfhydryl group to the wobble U34 [78], [79].

The next most persistent tRNA modification genes in *Mollicutes* are those coding for: i) MnmE and MnmG (formation of cmnm^5^U), both lost only in *M. suis*, ii) the two methyltransferases TrmL and TrmB catalyzing respectively the 2′*O*-ribose methylation of the wobble pyrimidine (C and cmnm^5^-containing U) and the formation of an m^7^G**^+^** (carrying a positive charge) in the extra arm (variable loop) of a large subset of tRNAs (position 46), both missing only in 6 non-culturable mollicutes (nodes 2 and 5 and **Figure 1B**), and iii) the site-specific TruB catalyzing the formation of ψ in all tRNAs, missing in the three hemoplasmas (node 5) and in *M. genitalium* and *M. pneumoniae* (node 6). Except for m^7^G**^+^** at position 46 in the variable loop and ψ at position 55 of the Tψ-loop, these modifications are again located in the anticodon loop of tRNAs (wobble position 34, **Figure S3**). The MnmA/MnmE/MnmG and TrmL enzymes all play key roles by restricting the corresponding modified tRNAs in decoding only the 2 purine-ending codons of a 4-synonymous codon set, while m^7^G**^+^**46 and ψ55 allow stabilization of the L-shape 3D-conformation of all tRNAs [80].

The essential *E. coli* and *B. subtilis* tRNA-A34 deaminase (TadA, formation of the wobble inosine) is present only in species of the Spiroplasma and AAP groups (lost at node 28). The complete elimination of *tadA* was shown to be a stepwise process. It started with specific mutations in the active site of TadA, was followed by the lost of one tRNA^Arg^ (anticodon CCG) that became useless before the final loss of the *tadA* gene [81]. Similarly, the loss of the essential tRNA-lysidine synthetase TilS (k ^2^C34) in *M. mobile* and the three hemoplasmas (node 5) correlates with a compensatory C-to-U mutation at the wobble position 34 in the tRNA^Ile^ substrate. In the case of *M. mobile*, the mutant tRNA^Ile^ was shown to harbor an unmodified wobble U34 instead of the normal k^2^C34. Using *M. mobile* ribosome in a cell-free *in vitro* system, this mutant U34-containing tRNA^Ile^ was shown to decipher preferentially Ile-AUA codon but not when *E. coli* ribosome was used, suggesting changes in the mollicute ribosome. This decoding readjustment is also dependent on additional mutations in *M. mobile* IleRS, allowing the mutated enzyme to preferentially aminoacylate U34-containing tRNA^Ile^ [82]. In the case of *M. penetrans*, MetRS was shown to better discriminate between tRNA^Ile^-CAU and tRNA^Met^-CAU than the canonical bacterial MetRS [83]. These examples demonstrate the high plasticity of the various components of translation machinery subsequent to the elimination of experimentally determined essential genes in *E. coli* or *B. subtilis* such as TilS and TadA, while preserving the accuracy of the decoding process.

The less persistent tRNA modification enzymes are: the site-specific methyltransferase TrmK (m^1^A**^+^**22, also carrying a positive charge) missing only in the Hominis sub-group (node 27); the methyltransferase TrmN (alias TrmN6; m ^6^A37) missing in all species of the Pneumoniae sub-group (except *Ureaplasma parvum* and *U. urealyticum*) and in the six non-culturable mollicutes (nodes 2 and 5); the multi-site specific pseudouridine synthase TruA (Psi38–40) that is missing in all species of the Hominis sub-group (node 27) plus the three non-culturable hemoplasmas (node 5).

For the remaining tRNA modification enzymes, a few are retained in a small subset of *Mollicutes* (**Figure 1B**). These are the G-to-Q transglycosylase (Tgt) acting at the wobble position 34 of a subset of tRNAs and its associated enzymes QueA, QueG, all lost very early (nodes 2 and 37 in **Figure 3**), leaving only *A. laidlawii* with tRNAs possibly containing Q34. Out of three dihydrouridine synthases characterized in *E. coli*, only one is present in *B. subtilis* and in the Spiroplasma group and *A. laidlawii*. Likewise, isopentenyl transferase MiaA responsible for i ^6^A37 formation, MiaB plus MtaB responsible for the subsequent methylthiolation of i^6^A37 (ms ^2^i^6^A) and t^6^A37 (ms ^2^t^6^A) respectively, are lost several times independently in the majority of mollicutes.

With the exception of an *E. coli* TtcA homolog (s^2^C32 formation), possibly acquired by lateral gene transfer in *M. penetrans*, all modification enzymes present in *E. coli* and absent in *B. subtilis* are also absent in *Mollicutes.* Examples include MnmC (mnm^5^U34 from cmnm^5^U34), CmoA/CmoB (cmo^5^U34), SelU (seU34 and ges^2^U34 from s^2^U34), TmcA (ac^4^C34), TsaA (m^6^t^6^A37 from t^6^A37), TrmH (Gm18), TrmA (m^5^U54), TruC (ψ65) and TruD (ψ13). These modification enzymes obviously emerged in other phyla than the *Firmicutes*.

An interesting case concerns TrmFO catalyzing the folate-dependent methylation of the conserved uridine at position 54 (m^5^U54) in the Tψ-loop of tRNAs of Gram-positive bacteria [84]. Sequencing of tRNAs from *M. capricolum* and *M. mycoides* revealed the absence of m^5^U54 in tRNAs, while two and even three TrmFO homologs were found in the Spiroplasma sub-group (**Table S3**). Only one of the three isoforms is present in a few species of the Hominis sub-group and was probably inherited by lateral gene transfer (node 14 in **Figure 3**), possibly from another ruminant mycoplasma from the Spiroplasma sub-group [34]. The target specificities of the two TrmFO homologs in *M. capricolum* and *M. mycoides* while still to be determined, are obviously distinct from the *B. subtilis* tRNA-specific TrmFO, which illustrates again the evolutionary malleability of modification enzymes.

#### The special case of tmRNA

In addition to tRNAs, a transfer-messenger RNA (tmRNA) and its associated protein SmpB have also been identified in all mollicutes. Their function is to rescue stalled ribosomes during translation. This tmRNA folds into a tRNA-like domain (TLD), that shares many structural and functional similarities with tRNAs. In particular, the UUC sequence of the T-arm loop of *E. coli* tmRNA is post-transcriptionally modified into m^5^UψC. The m^5^U residue is introduced by the S-Adenosyl-L-Methionine-dependent TrmA and the ψ probably by TruB [84]. As mentioned above, TrmA is missing in all *Mollicutes* and the function of TrmFO in these organisms is still unclear. The pseudouridine synthase TruB, present in many mollicutes (see above), could therefore also catalyze ψ formation in the *Mollicutes* tmRNAs.

#### Ribosomal RNA modification enzymes

Many bases and riboses of rRNAs are post-transcriptionally modified like in tRNAs (**Figure S3**). Most modifications are introduced during pre-rRNA maturation and ribosome assembly, and just a few are formed at the level of the 30S and 50S subparticles or of the entire 70S ribosome. The conservation and clustering of modifications in the decoding center of the 30S subunit and in the peptidyl-transferase center of the 50S subunit, attests their important roles in the translation process.

Out of the total 33 genes coding for rRNA modification enzymes in both *E. coli* and *B. subtilis*, only 19 remain in *Mollicutes*, and only four are ubiquitous (**Figure 1A**). These are: i) the region-specific RsmA, catalyzing the dimethylation of two adenines at positions 1518 and 1519 (m^6,6^A, *E. coli* numbering) of helix 45 located close to the decoding site in 16S rRNA, ii) the site-specific RsmH catalyzing, the formation of m^4^C1402 of helix 44 at the P-site of the 30S subunit, iii) the multi-site specific RluD, catalyzing the isomerization of uridine into pseudouridine at three neighboring positions (1911, 1915 and 1917, *E. coli* specificity) of helix 69 in 23S rRNA, and iv) the site-specific RlmB, catalyzing the methylation of 2′-hydroxyl group of G2251 (Gm) in the P-loop (helix 80) of 23S rRNA (**Figure S3**). The ubiquitous m^4^C1402 of helix 44 of 16S rRNA can be further methylated on the ribose into m^4^Cm1402 by RsmI, an enzyme found in all *Mollicutes* except the three hemoplasmas (node 5 in **Figure 3**), whereas the ubiquitous ψ1915 of helix 69 in 23S rRNA can be further hypermodified into m^3^ψ by RlmH only after the 70S ribosome is formed, thus at very late stage of ribosome assembly. RlmH is lost in the hemoplasmas (node 5), the three phytoplasmas (node 2), and three of the six members of the Pneumoniae group (node 7). In the 3D-architecture of the ribosome, this hypermodified helix 69 extrudes from the 50S subunit toward the decoding center of the 30S subunit, close to helices 44 and 45, where the other universally conserved multi-modified rRNA sequences are located.

Among other fairly persistent genes are those encoding RluC catalyzing the isomerization of U955, U2504 and U2580 into pseudouridines, two of which belong to the peptidyl transferase center (PTC)-loop of 50S subunit, and RsmG catalyzing the formation of m^7^G**^+^**527 (carrying a positive charged on methylated *N7*) in helix 18 of the decoding center of 30S subunit. RluC is absent only in the three phytoplasmas (node 2) and RsmG is absent in *Ca*. Phytoplasma mali and in the hemoplasmas (node 5, **Figure 3**).

Many rRNA modification enzymes are lost in a large group of *Mollicutes* but with different patterns (**Figure 1B**). RluB catalyzing the formation of ψ2605 in helix 93 of the peptidyl-transferase center and RsmD catalyzing the formation of m^2^G966 in helix 31 of the decoding center are both absent in the group Pneumoniae (lost at node 12). Whereas, RsmB, catalyzing the formation of m^5^C967 located next to m^2^G966 mentioned above, is present in all species of the Spiroplasma sub-group and absent in all species of the Pneumoniae, Hominis and AAP sub-groups (loss at nodes 3 and 28). RsmE, catalyzing the formation of m^3^U1498 nearby the conserved m^4^Cm1402 in helix 44 of the decoding center of 16S rRNA, is present in all species of the Hominis sub-group and a few species of the Spiroplasma and Pneumoniae sub-groups. The case of RlmCD is special. It catalyzes the formation of m^5^U at two positions (747 in helix 35 and 1939 in helix 71) in 23S rRNA of *B. subitilis*, while in *E. coli* two paralogous enzymes (RlmC and RlmD) are needed to catalyze m^5^U747 and m^5^U1939 formation respectively [85]. RlmC/RlmCD is present in a few species of the Hominis sub-group only, while RlmD is absent in all mollicutes. Finally, RsuA, catalyzing formation of ψ516 in helix 18 of rRNA 16S, is present in *A. laidlawii* and few species of the Hominis sub-group only, whereas the dual t+rRNA specific RlmN (m^2^A2503 in 23S rRNA + m^2^A37 in tRNA, *E. coli* specificity) remains in only four mollicutes: *S. citri*, *Ureaplasma* spp., *M. penetrans* and *A. laidlawii* (**Figure 3**).

Two orphan RNA methylase genes are found in *B. subtilis* but absent in *E.coli*: Y*sgA*, encoding a putative TrmH/SPOUT-like 2′-*O*-ribose RNA methyltransferase (COG0566C) and renamed *rlmB2* because of its close relationship with *rlmB* catalyzing the formation of Gm2251 (see above) and *yqxC*, encoding an another similar FtsJ/Spb1/SPOUT-like 2′-*O*-ribose RNA methyltransferase. Because *B. subtilis* harbors a modified Gm2553 in the P-loop (helix 92, see **Figure S3**), for which the corresponding gene is unknown [86], we speculate that one of these two orphan genes correspond to the missing but important G2553-2′-*O*-ribose-rRNA methyltransferase, while the second one probably catalyzes 2′-*O*-ribose methylation at a yet unidentified nucleotide of RNA. Both YsgA/RlmB2 and YqxC are present in about half the *Mollicutes* analyzed but always present together (**Figure 1B**).

The *A. laidlawi* species seems to have conserved more rRNA modifications genes than other mollicutes. For example, RsmC (m^2^G1207) is found in *A. laidlawii* only (early loss at nodes 2 and 37 in **Figure 3**). Moreover, *A. laidlawii* harbors four RlmCD copies instead of only one in other *Mollicutes.* These enzymes should catalyze the formation of the six m^5^U identified in *A. laidlawii* 23S rRNA, their exact locations remaining to be determined [87]. The case of *E. coli* RluF (Psi2604) is special as no homolog is present in *B. subtilis* but it is found in *A. laidlawi*. Similarity search indicated that the closest homologs of the *A. laidlawii* RluF are homologs from Gram-positive bacteria other than *B. subtilis*, suggesting that *rluF* was either acquired laterally by *A. laidlawii* or lost in all the other *Mollicutes* and in *B. subtilis*.

The rational for the persistence of different sets of modifications in 16S and 23S rRNA in the different sub-groups of *Mollicutes*, is not obvious. Many of these rRNA modifications could ‘collectively’ contribute to optimizing ribosome biogenesis and/or translation process, different patterns of modified nucleotides being able to fulfill similar functions. In other words, the persistence of a gene coding for a given modified nucleotide in a mollicute may depend on which other genes were first eliminated during the genomic erosion, a situation similar to what geneticists call synthetic lethality.

#### Ribosome assembly, protein chaperones, helicases and protein modifications

In bacteria, the assembly of r-proteins onto precursor rRNA scaffolds to form functional 30S and 50S subunits requires over a dozen assembly/stability factors as well as post-translational protein-modifications. Ribosome assembly is a multistep process that can proceed through alternative pathways, ribosomal factors allow the favoring of one over the others, prevent kinetic traps, regulate ribosome assembly and stability, and introduce quality control steps (reviewed in: [88], [89], [90], [91].

The most important factors are the GTPases EngA (also named Der in *B. subtilis*), ObgE (also named CgtA or Obg in *B. subtilis*), not present in *E. coli* but widely distributed in Gram-positive bacteria), and the ATPase EngD (YyaF in *B. subtilis*). They stimulate and stabilize specific steps of 50S subunit assembly (or 70S in the case of EngD) and are ubiquitous in all *Mollicutes* (**Table S3**). Three additional GTPases are involved in maturation of the 30S or 50S subunits, they are also well preserved in *Mollicutes*: EngB (YeC in *B. subtilis*), EngC (also named RsgA, CpgA in *B. subtilis*) and Era (Bex in *B. subtilis*). EngB is missing only in the non-culturable *M. suis*, while EngC and Era are missing in the 3 hemoplasmas (node 5, **Figure 3**). Both EngC and Era bind to the 3′ end region of the small rRNA, to helix 44 and to penultimate helix 45, respectively [92], [93]. In *B. subtilis*, EngC is phosphorylated at many positions by a Ser/Thr kinase/phosphatase pair PrkC/PrpC, the same enzymes that phosphorylate elongation factor EF-Tu. PrkC and PrpC are present in all members of the Spiroplasma and Pneumoniae sub-groups and in a few species of the Hominis sub-group but totally absent in AAP species, attesting that EngC and EF-Tu phosphorylation, probably regulatory devices [94], are not essential. RbgA and YqeH are two GTPases found only in Gram-positive bacteria. RbgA functions by interacting with the precursor 45S ribosomal subunit lacking r-proteins L16, L27 and L36 [95], while YqeH acts on the pre-assembly 30S subunit [96]. Only homologs of RbgA are found in all mollicutes, while homologs of YqeH are found only in all species of the Spiroplasma group, in a few species of the Pneumoniae group, and in *A. laidlawii*. HflX is an important bacterial multifunctional RNA-binding protein belonging to the GTPase ObgE/CtgA superfamily [97]. It allows small RNA base-pairing with other RNA and facilitates mRNA degradation and polyadenylation-mediated RNA decay. Despite its conservation in a majority of bacteria, it is present only in *A. laidlawii*.

RbfA is a cold shock-response, non-GTPase ribosome-binding factor that acts on pre-30S subunit containing 17S rRNA and is required for an efficient processing of the 5′ end of 17S rRNA [98]. This assembly factor is present in all *Mollicutes*. In contrast with this ubiquitous RbfA, two other non-GTPase ribosome maturation factors, RimM and RimP that act at late step of 30S assembly, before the RbfA/EngC/Era checkpoints (see above and [99]), are found only in a few *Mollicutes*. The 50S binding protein YbhY/YqeI, present in both *E. coli* and *B. subtilis*, is missing in all *Mollicutes* Only the *B. subtilis* ribosome binding proteins YaaA has homologs in species of the Hominis and Pneumoniae sub-groups. The ribosome modulation factor RimF and the two ribosome associated proteins YibL, YjgA, all absent in *B. subtilis*, are also absent in all mollicutes (**Table S3**).

To be functional, proteins involved in ribosome biogenesis and translation must correctly fold. This quality control activity depends on a network of chaperone systems, among them are the DnaK (ATPase-Hsp70) and DnaJ (Hsp40), acting with its co-chaperone nucleotide exchange factor GrpE. This multiprotein machinery is present in all mollicutes. In addition to the ribosome associated chaperone Tig factor mentioned above, an alternative cytoplasmic, non-ribosomal associated chaperone complex GroEL(ATPase-Hsp60)/GroES [50] always exist in bacteria. However at variance with the ubiquitous DnaK-dependent systems machinery and the almost ubiquitous Tig system (lacks only in *M. suis*), multimeric GroEL/GroES complexes exist in only a few mollicutes, including *S. citri*, all AAP species, and a few species of the Pneumoniae group. Moreover, the other ribosome-associated heat-shock protein Hsp15 (HslR/YrfH) present in most bacteria is absent in *Mollicutes*, except in *A. laidlawii* (at node 38 in **Figure 3**). Of the five DEAD-box RNA helicases identified in *E. coli* (SrmB, DbpA, DeaD, RhlE, RhlB) and four in *B. subtilis* (CshA, CshB, DeaD, and YfmL) [100], [101], none, one, or maximum two helicases are found in *Mollicutes* (**Table S3**). Because nucleic acids in *Mollicutes* have low G+C contents (**Figure S1**), energetically costly ATP-dependent RNA helicases required to remodel certain RNA domains and facilitate peculiar RNA-protein interactions might have become obsolete.

Lastly, post-translational modifications of selected residues occur in a few r-proteins. In *E. coli* and/or *B subtilis*, L11 is methylated by PrmA, and S5, S18 and L12 are acetylated (the acetylated form of L12, being named L7) by RimJ, RimI and RimL respectively. RimK and PrmB catalyze the addition of glutamic acid residue to the C-terminus of S6 and L3 respectively. RimO and its associated co-factor YcaO catalyze the addition of a methylthio group to an aspartic residue of S12, a process that depends on a sulfur relay system [102]. Of all these protein modification enzymes, only RimI, RimL and RimK remain in just a few mollicutes (**Table S3,** **Figure 1B**). For the acetyltransferase RimI, the evolutionary scenario is complex with many predicted losses and a potential acquisition by lateral gene transfer (LGT) in *M. fermentans*. The case of RimK is also interesting as it is found only in *M. genitalium* and *M. pneumoniae*, which also suggests a LGT event. It would be interesting to understand why these two protein modification enzymes RimI and RimK had to be recovered along the genomic erosion path of the *Mollicutes*

Not only r-proteins but also translation factors are post-translationally modified. Release factors, RF1 and RF2 of *E. coli* are methylated at a glutamine residue of the universally conserved GGQ motif by the methytransferase PrmC (initially named HemK) in *E.coli* [103]. A close ortholog of PrmC exists in *B. subtilis* and majority of mollicutes, except in the 3 phytoplasmas (node 2, **Figure 3**). One conserved lysine residue of *E. coli* elongation factor EF-P, is modified to β-lysyl-lysine by the YjeK, YjeA (PoxA), and YfcM proteins [104], [105]. In *B. subtilis*, a homolog of YjeK exists but not of YjeA and YfcM, suggesting that *B. subtilis* EF-P is not modified. None of the mollicutes analyzed contain homologs of these EF-P modification enzymes.

#### RNA processing/Ribonucleases

The various RNA components of the bacterial translation machinery are synthesized as longer precursor molecules that require subsequent processing steps, sizing, and 5′ or 3′ ends trimming by a combination of endo- and exo-nucleases. These ribonucleases also play an important role in controlling the activity and quality of the translation machinery and the regulation of gene expression by RNA turnover. RNases generally harbor broad, sometimes overlaping specificity with other RNases, making difficult to determine their intrinsic essentiality. Also, at variance with the six other categories of proteins analyzed above, the set of RNases in Gram-negative and Gram-positive bacteria are quite different, some RNases are essential in one organism but not in the other [106], [107].

Of the 27 genes coding for RNases and related proteins we analyzed, only three were found in genomes of all *Mollicutes*: the two components of RNase P, the ribozyme (RnpA, M1-RNA) and its C5 protein component (rnP), and the two endonucleases J1 and J2 (**Figure 1A**). RNase P is a universally conserved metallo-ribonucleoprotein-type of endonuclease (also called 5′-tRNAse) that specifically removes the 5′-leader sequence of pre-tRNAs, pre-tmRNA and pre-4.5 S RNA of the protein secretion pathway to produce mature 5′-termini [108]. RNase J1 (RnjA) and its paralog RNase J2 (RnjB) are two enzymes present only in Gram-positive bacteria. They essentially play the same role as endonuclease RNase E (RnE) in Gram-negative bacteria. These endonucleases cleave single-stranded regions of various pre-RNA transcripts. However, a major difference with RNase E is that both paralogs RNase J1 and RNase J2 also catalyze the 5′-to-3′ exonucleolytic degradation of a large variety of 5′-phosphate containing RNAs [109], [110]. If this also applies to RNases J1/J2 of *Mollicutes*, this could explain in part the dispensability of a few other exonucleases during genome erosion (see below). Moreover, a large mRNA degradosome involving RNases J1 and J2, such as the one present in *B. subtilis* [111], [112], is lacking in *Mollicutes* because of the absence in many species of the genes encoding the endoribonucleases Y (RnY, node 27), the endonuclease M5 (RnmV), and the polyribonucleotide phosphorylase (PNPase, pnp, pnpA) (**Figure 1B**). A similar situation exists with the endoribonuclease RNase BN/Z (also called 3′-tRNase). This enzyme cleaves the 3′-tail of pre-tRNA transcripts to generate substrates ready for addition of the essential CCA sequence catalyzed by CCAase (see above). Since the 3′-CCA-end is encoded in all tRNA genes of *Mollicutes*, both RNase BN/Z and CCAse are not required, eliminating them avoids a futile cycle of removal and re-addition of these essential residues [113].

Three additional RNases are found in almost all *Mollicutes.* These are the double strand-RNA specific endoribonuclease III (RNase III or RnC), the single-strand specific 3′-to-5′-exoribonuclease RNase R (RnR), and the newly identified *E. coli* 3′-to-5′-exonuclease YbeY (YqfG *in B. subtilis*) [114]. RNase III and YbeY/YqfG are missing only in *M. suis*, whereas RNase R is missing only in the phytoplasmas (node 2, **Figure 3**). RNase III, is the only enzyme involved in sizing RNA precursors within their double-stranded regions [115], while RNase R and YbeY/YqfG remove the 3′-tails of tRNA and 16S-rRNA precursors, respectively. RNase R of *M. genitalium* removes the 3′-trailer in pre-tRNA in only one step [74], a process requiring an interplay of multiple enzymes in other bacteria. Thus because RNase R has become more selective during *Mollicutes* genomic erosion, several other RNase encoding genes have become dispensable. The missing RNase R in the phytoplasmas is probably compensated by the presence of a remaining exonuclease with similar specificity, such as 3′-to-5′-PNPase precisely found only in phytoplasmas and the single *S. citri* or RNase YhaM [116] present also in all phytoplasma species and in the Spiroplasma group (node 28).

An analogous situation exists for multivariants Ribonucleases H (HI = RnHA, HII = RnHB and HIII = RnHC) that cleave RNA of RNA-DNA hybrids. Their primary function is to prevent aberrant DNA replication at sites other than oriC. All *Mollicutes* contains at least one of the three isovariant RNases H (**Table S3**). Again, reducing the multiplicity of RNases harboring similar or overlapping specificities, while maintaining an essential cellular function, allows genomic downsizing.

Whereas Gram-negative bacteria possess only one essential oligoribonuclease (nano-RNase, Orn) for degrading oligoribonucleotides of 2–5 residues in length, *Firmicutes*, including *B. subtilis*, possess two non-orthologous nano-RNAses with redundant specificity: NrnA (Ytql) and NrnB (YngD) [117]. All mollicutes, except *A. laidlawii* lack NrnB, but harbor one to three NrnA isozymes (**Table S3**). Interestingly, one of the extra *M. pneumoniae nrmA* gene (Mpn140) displays a pAp-phosphatase activity with the production of AMP and orthophosphate [118]. This unexpected multiplicity of nano-exonucleases with redundant specificities, coupled with the peculiar phosphatase activity for 3′-phosphoadenosine-5′-phosphate (pAp), is of advantage for *Mollicutes* that cannot synthesize RNA and DNA building blocks and thus require alternative solutions for scavenging nucleotide precursors.

An important *B. subtilis* pyrophosphohydrolase (Bsu-RppH), functionally analogous to *E. coli* Eco-RppH [119], is absent in the majority of *Mollicutes* but present in *A. laidlawii*. This RNA hydrolase catalyzes the removal of pyrophosphate from the 5′-end of nascent triphosphorylated RNA transcripts, a function that is probably fulfilled in mollicutes by RNase J1 (see above). Also, the Hfq-dependent mRNA decay machinery mentioned above and the MazF-dependent cleavage of 16S rRNA system [120], [121] were lost early during *Mollicutes* evolution with the exception of *A. laidlawii*. Lastly, *M. gallisepticum* was the first analyzed bacterium in which RNA was shown not to be polyadenylated [122], a feature that probably applies to all *Mollicutes*. RNase Bsn (yurI *in B. subtilis*) is an RNase of Gram-positive bacteria that remains in a few species of the Hominis sub-group. It hydrolyses RNA non-specifically into oligonucleotides with 5′-phosphate and probably plays a role in nutrient cycling. A few additional RNases, present in only Gram-negative bacteria are also absent in *B. subtilis* and all *Mollicutes* (**Figure 1C**).

### Defining a Minimal Protein Synthesis Machinery in *Mollicutes*

**Figure 4:**
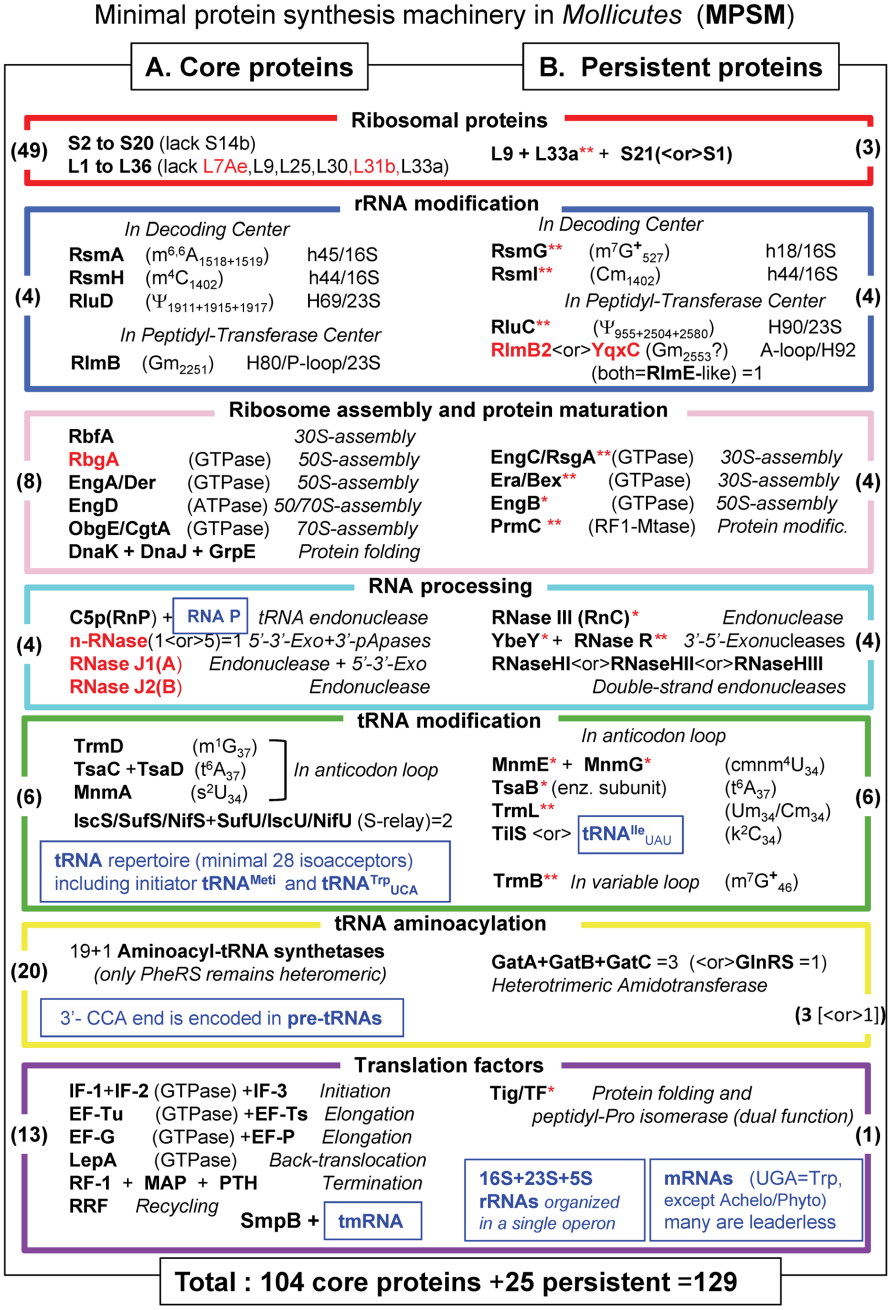
The minimal set of proteins for a functional translation apparatus in the 39 *Mollicutes* species. The acronyms of the 129 selected translation proteins in *Mollicutes* are divided in 2 parts: in A (left part), the 104 core proteins present in all *Mollicutes* analyzed are listed, while in B (right part) 25 additional proteins supposed to complement the 104 core protein are indicated. The acronyms and corresponding color code for the boxes are as in **Figures 1** and **3** and the corresponding names are given in **Table S2**). When the acronym in bold black letters is followed by one red asterisk, the proteins are absent in the non culturable *M. suis* and when followed by two red stars proteins are absent in the 3 hemoplasmas and/or the phytoplasmas (all these are present in panel B only). All numbers in brackets within boxes correspond to those indicated in part D of Figure 1. Acronyms indicated in red correspond to proteins that are found in *B. subtilis* and not in *E. coli*. The various types of translation-associated RNAs are indicated in small blue boxes. In the cases of tRNA and rRNA modification enzymes, the type of nucleotide modification and their positions in RNA as identified in *E. coli* are also given. Modified nucleotides m^7^G and m^1^A carries a positive charge at neutral pH (indicated by a +). X<or>Y means that either protein X or protein Y is found in mollicutes. However because of their overlapping functions or analogous specificities, the common essential function is preserved in all the 39 *Mollicutes* analyzed. The indication ‘n-RNases (1<or>5)’ means that one ancestral gene has been duplicated several times independently and each mollicute contain 1 to up 4 exemplars (they were however counted for one enzyme in our statistic). The average G+C % content in genome of the 39 *Mollicutes* analyzed is 27.6 varying from 21.4 in *Ca*. Phytoplasma mali to up to 40.0 in *M. pneumoniae* (Table S1).

The major goal of this work is to identify the minimal set of proteins that can sustain ribosome biogenesis and translation of the genetic code in self replicating bacteria with reduced genomes (MPSM for Minimal Protein Synthesis Machinery). Comparative genomics of 39 *Mollicutes* species allowed the identification of 104 genes encoding ubiquitous translation proteins designed as the core set herein. The acronyms of these proteins are listed according to their main functions in **Figure 4**. The majority of these core proteins are present in both *B. subtilis* and *E. coli*, the exceptions are proteins that are found only in Gram-positive bacteria (indicated in red; **Figure 4A**). In *M. genitalium* and *M. pneumoniae* almost all (except 4) of these 104 proteins were experimentally demonstrated to be essential (**Figure S2**), attesting their primordial importance for ribosome biogenesis and function in the context of *Mycoplasma* metabolism.

This set of 104 core proteins might not be sufficient for ribosome biogenesis and translation to work. Indeed, extant culturable *Mollicutes* maintain a set of translation proteins above an apparent lower limit of 138 (**Figure 2**). An additional set of essential proteins, not necessarily the same in each species, are obviously required. Among them are the 17 persistent gene products discussed above that are absent only in one (usually *M. suis*) or several non-culturable *Mollicutes* (indicated with red asterisks in **Figure 4B**). Eight additional proteins that are notably persistent or can only be replaced by an alternate mechanism have been added in the MPSM. These are: i) r-protein L9 (RplI) absent only in *M. penetrans* and three non cultivable species, L9 interacts with tRNA in the P site and limits mRNA slippage during translation; ii) r-protein S21 (PpsU) that is essential in the absence of r-protein S1 (RpsA), particularly for translating leaderless mRNAs; iii) 2′-*O*-RNA methyltransferase RlmB2 or YqxC predicted to methylate a conserved G residue in the A-loop (helix 92) of the peptidyl-transferase center of 23S rRNA (counted for one protein); iv) one of the three paralogous double-stranded endonucleases (RNases HI, HII, HIII) as all mollicutes harbour at least one of these enzymes that possibly could have broad specificity; v) the essential lysidine-tRNA transferase (TilS) that can be lost only if compensatory mutations occur in the tRNA recognition domain of IleRS and the anticodon of tRNA^Ile^; finally vi) the three subunits of the Gln-tRNA amidotransferase complex (GatA-GatB-GatC) of the Gln-tRNA amidotransferase complex essential for the formation Glutamine-tRNA^Gln^ in *Mollicutes* lacking the Glutamine-tRNA synthetase GlnRS (counted for 3 proteins).

Proteins that were easily lost during *Mollicutes* evolution were not included as essential elements of an MPSM (**Figure S3A**). However, some of these proteins may fine-tune ribosome biogenesis, improve efficiency of translation and/or display other side functions, such as coupling of translation with transcription and/or regulating protein expression. Finally, proteins that are absent in all *Mollicutes* were definitively discarded as elements of the MPSM, the majority of these are also absent in Gram-positive bacteria (**Figure S3B**).

Therefore, in absence of stress conditions that require specific proteins not discussed here, we propose that these 17+8 = 25 proteins, combined with the core of 104 proteins, comprise a theoretical MPSM of 129 proteins. This MPSM corresponds to a set of well characterized homologous proteins in our model bacterial systems and they are encoded by the most persistent genes in the *Mollicutes* analyzed. However, because some genes are still of unknown function in *E. coli*, *B. subtilis* and *Mollicutes*, we cannot exclude the possibility that a yet unidentified protein involved in the biosynthesis or function of the ribosome might have been missed.

Our evaluation of 129 minimal translation associated genes accounts for a large fraction of the total genes identified in mollicutes with reduced genomes (26% in the case of *M. genitalium* and 18% for *M. pneumoniae*). The protein synthesis factory is clearly the dominant and most energy consuming process in small cells such as *Mollicutes* [14].

The progressive reduction of the size of precursor RNAs (mainly mRNAs and tRNAs) by reducing their 3′ and/or 5′-tails is probably also part of the genomic size economization strategy. In *Mollicutes*, 18% of mRNA in average are leaderless mRNAs ([123], thus lacking the classical/canonical Shine-Dalgano (SD) sequence required for specific translation initiation on 30S subunit. Similarly precursor tRNAs have shorter 5′-leader sequence and no 3′-tail (see above). However, because of the constraint of maintaining canonical bacterial type of ribonucleoprotein 30S and 50S particles, the length of 16S and 23S rRNAs in *Mollicutes* is almost identical to those of other bacteria [124].

### Comparison with naturally occurring Minimal Protein Synthesis Machinery

**Figure 5:**
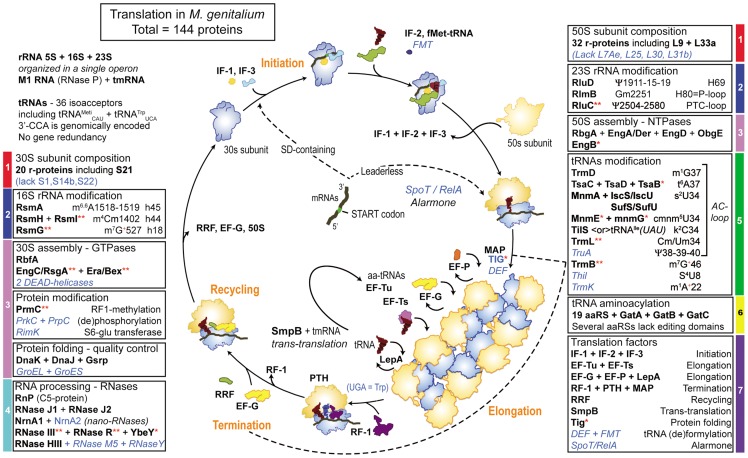
Schematic view of ribosome assembly and translation cycle in *M. genitalium*. In each box are indicated the acronyms of proteins encoded in the genome of *M. genitalium* (Table S3). The acronyms in black bold letters correspond to proteins listed in Figure 4 (A+B) of the minimal protein synthesis machinery (MPSM), only RlmB2<or>YqxC is missing (see text). When the acronym is followed by a red asterisk, the protein is absent in the non-culturable *M. suis* and when followed by double asterisks, proteins are absent in the 3 hemoplasmas and/or the phytoplasmas. The acronyms in italic blue letters correspond to proteins that are absent in many mollicutes, but present in *M. genitalium* and *M. pneumoniae*. The color codes for each box are the same as in Table 1. Steps of translation are indicated in orange. Elongation (ribosomes assembled on mRNA forming polysomes) and termination are indicated by a circle dashed line. The step corresponding to the action of RF-1 has been isolated from the rest of the polysome, for better visualization. Depending on whether an mRNA harbors a 5′-leader sequence with SD-sequence or is leaderless, initiation occurs either on 30S subunit or 70S ribosome respectively. This figure allows a direct comparison with the similar one for translation cycle in Bacteria versus Eukaryotes published by Melnikov *et al* from M. Yusupov's laboratory in Strasbourg, France [125].

The best-studied extant *Mollicutes* with reduced genomes and capable of independent growth are the two phylogenetically related *M. genitalium* and *M. pneumoniae.* With a total of about 482 CDS, including 144 CDS for the translation machinery, for a 0.580 Mbp genome, *M. genitalium* is generally considered as the best representative of a minimal free-living cell. A schematic view of the translation machinery in *M. genitalium* is depicted in **Figure 5**, together with the list of all the elements required for ribosome biogenesis and mRNA translation. The 128 proteins classified above as belonging to the MPSM are in bold-black acronyms (only the putative r-RNA modification enzyme RlmB2/YqxC of the selected 25 additional proteins is missing), while the additional 16 proteins present in *M. genitalium* are in blue italic acronyms (see also **Figure S4**). These latter proteins include two DEAD- box helicases, one protein kinase (PrkC) and its associated protein phosphatase (PrpC), one r-RNA protein modification (RimK) and two chaperones (GroEL+GroES), all classified as proteins of ribosome assembly and protein maturation. In addition are found three ribonucleases of the RNA processing (RNase M5, RNase Y and a second nano-RNase), three tRNA modification enzymes (TruA, ThiI and TrmK) and three translation factors (DEF, FMT, SpoT/RelA). These proteins, especially GroEL/GroES, RNase MV and RimK are lacking in many other *Mollicutes* (**Figure 1B** **, Table S3**), RimK is even absent in *B. subtilis* and arose in both *M. genitalium* and *M. pneumoniae* probably by lateral gene transfer (see above). In *M. genitalium*, these proteins may have specific functions such as fine-tuning of RNA processing and ribosome assembly, mRNA translation and its regulation in response to specific physiological demands of the cell. Despite these differences, the translation apparatus in *M. genitalium* fits well with the MPSM concept developed above and closely resembles the classical scheme of translation in bacteria [125].

The most remarkable features of protein synthesis in *M. genitalium* and other *Mollicutes* with minimal genomes are: 1) almost all canonical r-proteins are present (however, as shown in the case of *M. pneumoniae* [126] not all r-proteins may be present in every ribosome, a certain degree of plasticity in r-protein composition may exist according to specific type of mRNA to be translated); 2) the GTP/ATPases involved in 30S/50S/70S assembly are identical in sequence and number to those found in other bacteria with larger genomes, attesting that the assembly process follows a path extremely conserved in bacteria; the frequent lack of DEAD-box helicases probably results from the A/T-rich RNA sequences; 3) the DnaK-dependent protein folding/quality control system is ubiquitous. However in only a few *Mollicutes*, including *M. genitalium* and *M. pneumoniae*, GroEL/GroES are present and therefore should not be considered as essential; 4) the multiplicity of genes coding for nano-RNases allowing to scavenge for mononucleotide building blocks is of clear advantage for *Mollicutes* that are devoid of nucleotide biosynthetic pathway; 5) among post-translational protein modification enzymes, only the methyltransferase PrmC (HemK) that methylates termination factor RF-1 is conserved in *Mollicutes*; 6) a repertoire of 19 aaRSs plus the GatA/GatB/GatC amidotransferase complex allowing to generate Gln-tRNA^Gln^ and a minimal set of 28 isoacceptor tRNAs are used to decode all 62 sense codons into 20 canonical aminoacids; 7) an extra tRNA^Trp^ harboring an anticodon U*CA reads UGA as Trp [55], the absence of termination factor RF-2 being consistent with this scheme; 8) the methionine residue attached to initiator tRNA^Met^ is formylated in *M. genitalium* but in most mollicutes the formylation/deformylation enzymatic system (FMT/MAP) is absent and therefore not essential; 9) the majority of post-transcriptional enzymatic modifications in tRNA and rRNA are restricted to a few nucleotides located mostly in the anticodon loop of tRNA, the ribosomal decoding sites (h18, h44 and h45) of 30S subunit and the peptidyl transferase site (H90, H69) of 50S subunits; 10) the majority of the essential bacterial factors are needed, except the stress rescue and silencing factors TypA, AraFA and RsfA; 11) the SpoT/RelA alarmone system is present in *M. genitalium* and most species of the Pneumoniae sub-group but absent in all species of the Hominis sub-group; 12) tmRNA and its associate protein SmpB of the trans-translation system and the ribozyme RNaseP with only one associated protein RnP are preserved; 13) because of the use of numerous leaderless mRNAs in *Mollicutes*, an alternative mechanism of translation initiation exists beside the canonical Shine-Dalgano (SD)-depending mRNA initiation, translation initiation of SD-containing mRNA occurs on 30S subunit and is usually mediated by r-protein S1, while S1 but not S21 become dispensable for translation of leaderless mRNAs on intact 70S ribosome [39]; finally, 14) because of their small sizes, a *Mollicutes* species like *M. pneumoniae* contains only 140–200 ribosomes per cell volume of 0.067 µm^3^ [11], while an *E. coli* cell of about 1 µm^3^ usually contains several thousands of ribosomes [127].

### Concluding remarks and future prospects

This study shows that comparative genomics analyses can help define the minimal set of genes required for translation in *Mollicutes*. Translation genes that have not been lost in any of the species analyzed belong to a translation core that is most certainly needed to sustain protein synthesis. However, loss of a specific protein or enzyme in a given *Mollicutes* species does not necessarily translate in loss of the corresponding cellular function, as some cellular enzymes or proteins may display overlapping specificities or fulfill closely related, analogous functions. Occasional gene gains are also indicative of the need for compensation for the gene losses or acquiring new functionalities to maintain a reduced, but coherent functional protein synthesis machinery. The corollary of these premices is that different solutions to minimize translation machinery can evolve in different *Mollicutes* and it is illusory to try to define a universal minimal set of translation proteins that would be common to very distantly related bacteria (discussed in [28]).

The class of *Mollicutes* is particularly suited for defining a minimal translation apparatus. Not only do they include organisms that have eliminated many primordial metabolism genes (including translation genes), while retaining the capability to replicate and translating mRNAs in an axenic medium, but they also appear as some of the most evolved prokaryotes able to sustain complex metabolism with a minimum elements of its cellular chassis (discussed in: references [3], [4], [9], [10], [11]). Recent studies from independent laboratories have shown that two *Mollicutes* species (*Mesoplasma florum* and *Mycoplasma gallisepticum*) exhibit the highest known rate of base-substitutional mutation for any unicellular organism showing these are fast-evolving bacteria [69], [71]. Although *Mollicutes* species share a small genome size, our study indicates that there remains room for diversity even in a highly conserved apparatus such as translation. On one side of the spectrum, *M. suis* probably stands out as the most minimal organism with only 116 proteins dedicated to translation. At this stage, it is not understood how this uncultured organism that lives associated to red blood cells of its mammalian host is able to synthetize proteins with a machinery that appears so deficient. It is tempting to hypothesize that translation in *M. suis* requires factors from its host, but owing to the lack of general knowledge on hemoplasma biology, it is too speculative to further elaborate. On the other side of the spectrum, *A. laidlawii* has a much larger repertoire of proteins implicated in translation (183) than most other *Mollicutes* species, but still lower proteins than in our model bacteria *E. coli* (228) and *B. subtilis* (210). In fact, this species with other *Acholeplasmatales* also stands apart from other *Mollicutes* because it has larger metabolic capacities and is ubiquitous, being able to live as a saprophyte in soil, compost or wastewaters [128]. The reconstruction of the evolution of translation-related gene set in *Mollicutes* (**Figure 3**) indicated that *A. laidlawii* is probably the species among the *Mollicutes* that is the closest to the common ancestor with the *Firmicutes*.

Important aspects of genome downsizing in bacteria concern the accuracy, efficiency and regulation of the minimalist translation process. Recent works at studying aminoacylation of tRNA *in vitro* demonstrated that several aminoacyl-tRNA synthetases of *M. mobile* are prone to mistake the amino acid or the tRNA substrate to be charged (discussed above). Such mis-aminoacylations will lead to subsequent incorporation of wrong amino acids into proteins and consequently will reduce the global fitness of the proteome. The possibility that mis-incorporation of amino acids into the nascent polypeptide also occurs because of mis-functioning of the minimalist ribosome cannot be discarded [82]. Elimination of abnormal/misfolded proteins by the usually abundant cellular GroEL/GroES and/or DnaK-dependent chaperone/degradation system acting as promiscuous buffer of genetic variations should not be underestimated (see for example: [129]). As long as the remaining mutant proteins allow cell viability, a low quality of the proteome may even be of some advantage by contributing to the antigenic variation of the mycoplasma exposed to its host's immune response [70], [130].

The genome-scale analysis of soluble complexes in *M. pneumoniae* has revealed an unexpected high level of protein interaction leading to an estimate of some 200 molecular machines [11]. The ribosome assembly represents one of the most complex networks of interaction. Interestingly, among the 13 polypeptides for which a function was not yet attributed in this specific network, two of them were predicted in our analysis as DEAD-box RNA helicase (MPN623) and as endonuclease M5 (RnmV; MPN072); see **Table S3**. In fact, MPN623 was curated as an ATP-dependant RNA helicase in the work of Kuhner *et al* [11], which is consistent with our predictions.

The small number of proteins of the MPSM in *Mollicutes* is also reminiscent of the translation machinaries in mitochondria and bacterial endosymbionts [131]. However, in the case of mitochondria, a more massive gene and protein loss occurred, resulting in the loss or transfer to the nuclear host genome of majority of bacterial proteins encoding essential genes, including those related to protein synthesis machinery. Of the original bacterial machinery for translation, only genes coding for the structural RNA (t/r/mRNAs), have been preserved (only 16 Kbp in mammalian mitochondria). All the proteins required for the extant/modern mitochondrial ribosome assembly and translation are nuclear encoded, synthesized on the cytoplasmic ribosomes of the cell host, and subsequently imported into the mitochondria via several transport machineries. Despite this unique mitochondrial organization, translation in mitochondria is essentially bacterial-like. One major difference with *Mollicutes*, even with *M. genitalium* described above, is that only a small number of mito-mRNAs (mono- and di-cistronic) are translated, all coding for proteins that are part of the membrane reaction centers of the respiratory chain complexes. Consequently, all mito-ribosomes are permanently tethered to the inner membrane and its composition, especially around the polypeptide exit tunnel, is much different from bacterial ribosome. This peculiarity allows a better coordination of the synthesis of the highly hydrophobic mitochondrial proteins and their immediate assembly within the mitochondrial membrane [132]. The possibility exists that, beside the cytoplasmic ribosomes producing mainly soluble cellular proteins, a minor fraction of such specialized membrane-bound ribosomes also exists in *Mollicutes*, a cellular strategy that certainly allows better efficiency of certain membrane proteins. Another difference is that all mito-mRNAs are leaderless, while in *Mollicutes* the majority of mRNAs (80% in average [123]) harbor a Shine-Dalgano (SD) sequence that determines the translation initiation pathway followed (**Figure 5**). Beside these mitochondrial specifications, both organelles and mycoplasmas, uses UGA codon for Trp and the translation factors are essentially the same (except for the lack of mito IF-1), attesting for a very similar translation mechanism as depicted for *M. genitalium* in **Figure 5** (reviewed in: [133], [134], [135]).

Bacterial endosymbionts like *Wolbachia* (range of genomesize: 958–1,482 Kbp), and *Buchnera* (422–1,502 Kbp) that infect arthropods and aphids respectively have also evolved in a parasitic life-style by reducing their genome sizes. In some species such as *Carsonella ruddii*, *Candidatus* Tremblaya and *Nasuia deltocephalinicola*, the genomes are even smaller (160–112 Kbp). These tiny bacteria originated about 200 My ago from independent lineages of diverse bacterial groups. At variance with majority of *Mollicutes*, they cannot be cultivated as free-living organisms and live in a close symbiosis within the host cell, like an organelle. Beside nutrient exchanges, possible protein exchanges between the endosymbiont, the cell host and often cohabiting additional distinct co-endosymbiont(s) remain a matter of debate [136], [137]. Therefore, insect endosymbionts represent a heterogeneous group of organisms and those with the smallest genomes are not ideal model organisms to identify minimal gene sets for autonomous replication. However, examination of the available information on translation genes from a selected set of endosymbionts [138], [139] reveals that most persistent translation machinery genes in these minimal organisms correspond to a large part of the MPSM defined in *Mollicutes* (see **Figure S5**). However, from the smallest sets of endosymbiotic proteins it is difficult to build a self-constructing ribosome and successful translation machinery. Evidently in these cases additional proteins from the co-symbiont(s), the host mitochondria or even the host cell would have to complement those translation proteins of the endosymbionts.

Owing to the minimal size of their genomes, *Mollicutes* have been chosen as the starting point in efforts aiming at building a minimal cell using tools from synthetic biology (for review see [140]). The ambitious goal of these studies is not only to decipher all the functions required for sustaining a minimal life but also for building a cell chassis that could be used in biotechnological processes. Following major progress in DNA assembly, genome engineering and transplantation, this goal seems to be within reach. However, building a minimal cell requires an in-depth knowledge of the cell machinery including of the translation apparatus. Our results should contribute to this goal by providing not only one scenario for the MPSM, but rather a series of possible sets based on the analysis of the different *Mollicutes* sub-groups. This prediction is now open to experimental verification using synthetic biology.

## Materials and Methods

### Phylogenetic reconstruction

The phylogenetic tree required for the reconstruction of the ancestral gene sets at the different stages of *Mollicutes* evolution was generated using concatenated multiple alignments of selected 79 orthologous protein sequences. Proteins encoded by single copy genes present in the genome of all mollicutes were selected. This list is provided in the **Figure S1**. Multiple alignments were generated using MUSCLE [141], concatenatedusing Seaview [142] and curated from unreliable sites with GBlock [143]. The final concatenated alignment contained 10,686 sites. The phylogenetic tree was constructed by the Maximum Likelihood method using PhyML [144] available on the web server Phylogeny.fr [145]. The list of mollicutes analyzed with some of their genomic characteristics is given in **Table S1**.

### Mining genes encoding proteins of the translation apparatus in *Mollicutes*

The whole set of proteins of the of *Escherichia coli* str. K-12 substr. MG1655 and of *Bacillus subtilis* subsp. *subtilis* str. 168 translational apparatus were obtained from the Modomics [146], Biocyc [147], SEED [148], SubtiList [27] databases, and Kyoto Encyclopedia of Genes and Genomes [149], plus an extensive review of literature (**Table S2**).

Homology between *E. coli* and *B. subtilis* proteins was inferred by sequence similarity using a reciprocal BLAST search approach (bidirectional best hit). All *E. coli* and *B. subtilis* proteins were used as queries for BLAST searches in 39 selected genomes from distinct *Mollicutes* species included in the MolliGen genome database ([150]; http://www.molligen.org) (**Table S3**). In this database, initial annotated genomes were obtained from GenBank files. These genomes were further curated by expert annotation that resulted in changes in the functional annotation of specific CDSs and in adding CDSs that were missing in the initial Genbank file. This step of data curation was performed in the frame of the present project for all the homologs involved in translation. Multiple genomes from the same species were excluded from our dataset because initial analyses indicated that no intra-species differences are evident in the gene sets encoding proteins involved in a central process such as translation and ribosome biogenesis. They were nevertheless useful for confirming the presence or absence of a given gene or solving some abnormalities due to occasional sequencing errors in the dataset. BLASTp searches were first conducted with an e-value cutoff of e^−8^. However, proteins sequences retrieved with an e-value ranging from e^−8^ to e^−3^ were maintained in the dataset if a domain related to the considered query was detected using the Conserved Domain search engine [151]. When no hit could be found for a given protein query in one of the *Mollicutes* genomes, the protein of the closest species identified as a putative hit for this query was used as a query for additional BLASTp and tBLASTn searches. For each query, sequences of the putative *Mollicutes* homologs were aligned with Clustal W [152]. Subsequent phylogenetic analyses were conducted by using the Neighbour Joining method in Mega5 [153]. Annotation of paralogs was resolved, when possible, by analyzing the microsynteny in MolliGen and the topology of the corresponding phylogenetic trees.

### Reconstruction of the ancestral gene set

The translation-related gene set at ancestral stages of *Mollicutes* evolution was inferred using probabilistic and parsimony approaches implemented in the COUNT software package [36]. We used the above described phylogenetic tree and a presence/absence matrix describing the occurrence of 210 genes over 39 *Mollicutes* genomes and one reference genome, *B. subtilis*. The posterior probabilities were calculated using a birth-and-death model. We maximized the likelihood of the data set using a gain–loss model with a Poisson distribution at the root. Gain rate for *B. subtilis* was fixed at 0 to avoid false prediction of many gene gains by this species. Several combinations of parameters were tested to maximize the likelihood. The best value was obtained with the edge length, loss and gain rates set at 4 gamma categories. Edge length and loss rate parameters had more impact than gain rate on the final likelihood of the optimized model. Wagner parsimony [37] was also used to infer ancestral gene sets. A gain penalty of 4 was used to minimize predicted gene gain events, in accordance with the massive genome reduction context of *Mollicutes* evolution.

## Supporting Information

Figure S1 Phylogenetic tree of the 39 selected *Mollicutes* species. The phylogenetic tree was generated using concatenated multiple alignments of selected 79 orthologous protein sequences, encoded by single copy genes present in the genome of all *Mollicutes* were selected. The corresponding list is provided below. Multiple alignments were generated using MUSCLE [141], concataned using Seaview [142] and further cured from unreliable sites by GBlock [143]. The final concatenated alignment contained 10,686 sites. The phylogenetic tree was constructed by the Maximum Likelihood method using PhyML [144] available on the web server Phylogeny.fr [145]. Concataned protein sequences were from the following 79 core genes: *rplA*, *rplB*, *rplC*, *rplD*, *rplE*, *rplF*, *rplJ*, *rplK*, *rplL*, *rplM*, *rplN*, *rplO*, *rplP*, *rplQ*, *rplR*, *rplS*, *rplT*, *rplW*, *rplX*, *rpmB*, *rpmC*, *rpmF*, *rpmH*, *rpmI*, *rpmJ*, *rpsC*, *rpsD*, *rpsE*, *rpsF*, *rpsG*, *rpsH*, *rpsI*, *rpsJ*, *rpsK*, *rpsL*, *rpsM*, *rpsNA*, *rpsO*, *rpsP*, *rpsQ*, *rpsS*, *rpsT*, *alaRS*, *asnRS*, *aspRS*, *cysRS*, *gltX*, *glyS*, *hisRS*, *ileRS*, *leuRS*, *lysS*, *metRS*, *pheS*, *serRS*, *thrRS*, *trpRS*, *tyrRS*, *rsmA*, *mnmA*, *trmD*, *tsaD*, *dnaK*, *engA/der*, *engD*, *rbfA/PB15*, *rbgA*, *IF-1*, *IF-2*, *IF-3*, *EF-P*, *EF-TS*, *EF-TU*, *RF-1*, *rrf*, *lepA*, *smpB*, *rnjA* and *rnp*. Number next to each species corresponds to Table S1. The six non-culturable *Mollicutes* are within a red dotted box.
(PDF)

Figure S2 Essential genes versus genes involved in translation. The essential genes are indicated on the right-hand site of the same panels A and B as in **Figure 1**; panel C is not shown as all the corresponding genes are missing all *Mollicutes* analyzed. An essential gene is indicated by a black background. NO, UK, NA apply to non-essential genes, to genes for which the essentiality is unknown and NA for genes that are missing (not applicable), respectively. In orange background are indicated the 17 proteins that are exclusively absent in one or several non-cultivable *Mollicutes* and considered as necessary for the MPSM. The data for *M. genitalium* are from Glass et al 2006 [16], for *M. pulmonis* from Dybvig et al 2010 [17], for *B. subtilis* from Kobayashi et al 2003 [154] and from data compiled on the Ecocyc database for *E. coli* [26].
(PDF)

Figure S3 Dispensable proteins of translation apparatus in Bacteria. General information is the same as in Figure 4, except that results are now divided into 2 main boxes: part A corresponds to the proteins that are easily lost during reductive *Mollicutes* evolution and part B corresponds to proteins that have not been found in any of the 39 *Mollicutes* analyzed. Proteins are classified according to the 7 categories defined in Table 1. Acronyms indicated in black italics letters correspond to proteins absent in many *Mollicutes* but found in both *E. coli* and *B. subtilis*; in red italic letters are proteins absent in *E. coli* and present in *B. subtilis*, whereas proteins present in *E. coli* and absent in *B. subtilis* are indicated in bold green letters. Several DEAD-box helicases exist in these two bacteria, while none or a maximum two of these helicases are found in the different *Mollicutes* (see Table S3). All numbers in brackets within boxes correspond to those indicated in part D of Figure 1.
(PDF)

Figure S4 Genes coding for proteins implicated in translation in *Mycoplasma genitalium*, in addition to the core set of proteins. The data and symbols (species numbering, acronyms and their corresponding color codes on the left, meaning of grey background within the table) are the same as those of Figure 1, part B entitled ‘Genes lost in some *Mollicutes* species only’. Data concerning the *M. genitalium* (species # 31) are boxed with yellow. All acronyms in bold letters on the left correspond to proteins that are present in *M. genitalium* (see in Figure S3) and also present in other *Mollicutes* (orange background) or in contrary absent in other *Mollicutes* (light green background). The MPSM (Minimal Protein Synthesis Machinery) of *Mollicutes* includes all the 24 proteins encoded by genes in the orange background (only RlmB2/YqxC is missing in *M. genitalium*). The light green background of the other acronyms small boxes (16 cases) means that the corresponding proteins do not belong to the MPSM, but the protein are present in *M. genitalium*.
(PDF)

Figure S5 Protein synthesis machinery in bacteria with reduced genomes. On the left part of the figure are listed the acronyms of the 129 proteins of the MPSM (Minimal Protein Synthesis Machinery) deduced from the comparison of genomes of 39 *Mollicutes* (see **Figure 4** and corresponding text in the main part of the manuscript). The central part of the figure is the common set of 111 proteins involved in the ribosome biogenesis and mRNA translation of 5 obligate bacterial endosymbionts of insects (*Buchnera aphidicola* strains BBp, Bap, BSg/618, 652, 653 Kbp respectively, *Candidatus Blochmannia floridanus/*710 Kbp and *Wigglesworthia glossinidia/*700 Kbp) and listed in Table 1 of the paper by Gil, Silva, Pereto and Moya [139]. On the left part of the figure is the common set of 97 translation proteins in 2 obligate insect symbionts (*Sulcia Muelleri*/190 Kbp and *Nasuia deltocephlinicola*/112 Kbp) cohabiting the same host cell *Macrosteles quadrilineatus*), listed in tables 2 and 3 of supplemental materials of the paper published by Bennett and Moran [138]. The acronyms and corresponding color code for the boxes are as in **Table 1** and **Figure 1** of the main text, the corresponding names being given in **Table S2**. Acronyms indicated in black bold letters correspond to proteins present in *E. coli* and *B. subtilis*, in bold red letters to proteins found in *B. subtilis* and not in *E. coli*, and in bold Green letters to proteins found in *Nasuia* only, not in the co-symbiont *Sulcia*. All numbers in brackets correspond to the total proteins found in each of the protein family boxes. The purpose of this comparison is to point out that the translation proteins identified as highly resistant to genomic erosion during *Mollicutes* evolution are the ones that are also resistant to genomic erosion in Insect endosymbionts. Moreover, when two obligate endosymbionts co-exist in the same host cell, some important proteins exist in only one of the two endosymbionts, attesting for probable functional complementation. A major difference between *Mollicutes* and bacterial endosymbionts is that the former can live in the absence of the host cell (they are self-replicative entities), while the latter are strictly dependent of the host cell, like an organelle. In other words, when an essential gene is lacking in the genome of an endosymbiont, one never sure whether the missing cellular function can be full fit (or not) by a ‘foreign’ protein originating from the co-endosymbiont, the host mitochondria and/or the host cell itself.
(PDF)

Table S1 List of selected (39) *Mollicutes* with some genomics and phenotypic (cultivability) features. The list of the 39 selected *Mollicutes* is given following the numbering used throughout the manuscript. The genomics features (Genome size, % G+C, #CDS) were obtained from Genbank. The data from the six non-cultivated *Mollicutes* are framed with a red dashed line.
(PDF)

Table S2 Proteins implicated in the biosynthesis and functions of translation machinery in bacteria. List of queries of *E. coli* and *B. subtilis* sequences used to search for homologs in *Mollicutes.* Accession numbers are from the NCBI Reference Sequence or UniProt databases. The product names for *E. coli* and *B. subtilis* proteins are from the Ecocyc and the Subtiwiki databases, respectively.
(XLSX)

Table S3 Table of putative homologs detected in the selected mycoplasma genomes. The orthologs for the queries either from *E. coli* or *B. subtilis* were searched as indicated in Material and Methods in the 39 selected *Mollicutes* When a hit was found its mnemonic was indicated in the corresponding cell of the table. When there was no homolog, the cell was left empty with a red background. In some cases, the ortholog in a given genome corresponds to a putative pseudogen; it is indicated as “pseudo” in the cell following the mnemonic(s). In some other cases, the *Mollicutes* gene seems to correspond to a fusion between the gene encoding the given homolog and another entity; it is indicated as “fusion” in the cell following the mnemonic. In some instances, the homolog was not found in the genome from the selected strain but was found in the genome(s) of other strain(s) from the same species; in that case the hit was considered positive and indicated “found in other strains” in the table. Finally, in some genomes that were not circularized, a homolog could be detected in a genomic region without an annotation; in that case, it was counted as positive occurrence and indicated “homolog” in the cell.
(XLSX)
