# Supplementary figures and images for: Predicting the Minimal Translation Apparatus: Lessons from the Reductive Evolution of Mollicutes
Source: PLoS Genet. 2014 May 8;10(5):e1004363. doi: 10.1371/journal.pgen.1004363 (PMC4014445; doi:10.1371/journal.pgen.1004363)

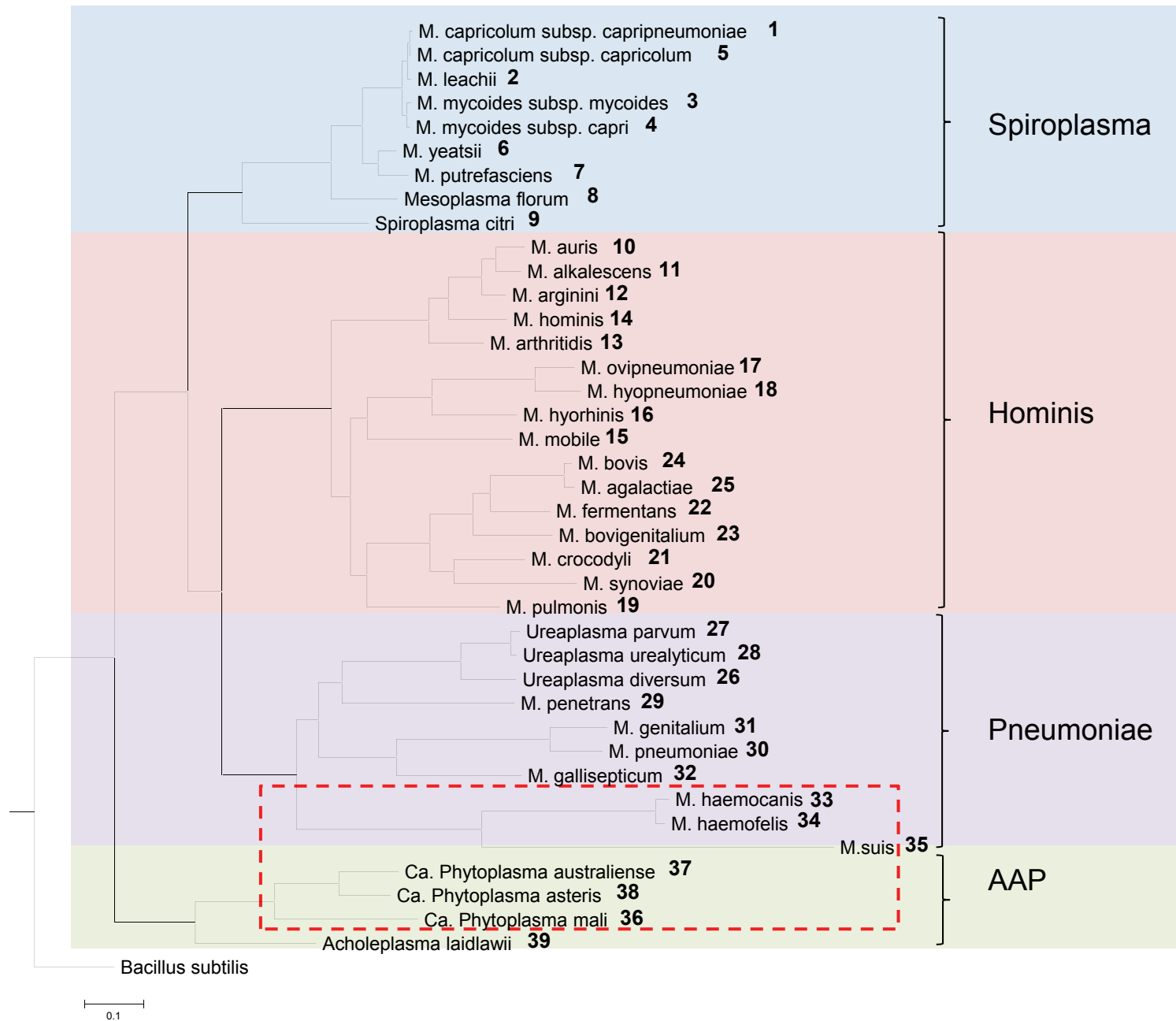

Supplement: Figure S1 — Phylogenetic tree of the 39 selected Mollicutes species. The phylogenetic tree was generated using concatenated multiple alignments of selected 79 orthologous protein sequences, encoded by single copy genes present in the genome of all Mollicutes were selected. The corresponding list is provided below. Multiple alignments were generated using MUSCLE [141], concataned using Seaview [142] and further cured from unreliable sites by GBlock [143]. The final concatenated alignment contained 10,686 sites. The phylogenetic tree was constructed by the Maximum Likelihood method using PhyML [144] available on the web server Phylogeny.fr [145]. Concataned protein sequences were from the following 79 core genes: rplA, rplB, rplC, rplD, rplE, rplF, rplJ, rplK, rplL, rplM, rplN, rplO, rplP, rplQ, rplR, rplS, rplT, rplW, rplX, rpmB, rpmC, rpmF, rpmH, rpmI, rpmJ, rpsC, rpsD, rpsE, rpsF, rpsG, rpsH, rpsI, rpsJ, rpsK, rpsL, rpsM, rpsNA, rpsO, rpsP, rpsQ, rpsS, rpsT, alaRS, asnRS, aspRS, cysRS, gltX, glyS, hisRS, ileRS, leuRS, lysS, metRS, pheS, serRS, thrRS, trpRS, tyrRS, rsmA, mnmA, trmD, tsaD, dnaK, engA/der, engD, rbfA/PB15, rbgA, IF-1, IF-2, IF-3, EF-P, EF-TS, EF-TU, RF-1, rrf, lepA, smpB, rnjA and rnp. Number next to each species corresponds to Table S1. The six non-culturable Mollicutes are within a red dotted box. (PDF) [file pgen.1004363.s001.pdf]
