# Supplementary material for: Predicting the Minimal Translation Apparatus: Lessons from the Reductive Evolution of Mollicutes
Source: PLoS Genet. 2014 May 8;10(5):e1004363. doi: 10.1371/journal.pgen.1004363 (PMC4014445; doi:10.1371/journal.pgen.1004363)
Supplement: Figure S2 — Essential genes versus genes involved in translation. The essential genes are indicated on the right-hand site of the same panels A and B as in Figure 1 ; panel C is not shown as all the corresponding genes are missing all Mollicutes analyzed. An essential gene is indicated by a black background. NO, UK, NA apply to non-essential genes, to genes for which the essentiality is unknown and NA for genes that are missing (not applicable), respectively. In orange background are indicated the 17 proteins that are exclusively absent in one or several non-cultivable Mollicutes and considered as necessary for the MPSM. The data for M. genitalium are from Glass et al 2006 [16], for M. pulmonis from Dybvig et al 2010 [17], for B. subtilis from Kobayashi et al 2003 [154] and from data compiled on the Ecocyc database for E. coli [26]. (PDF) [file pgen.1004363.s002.pdf]

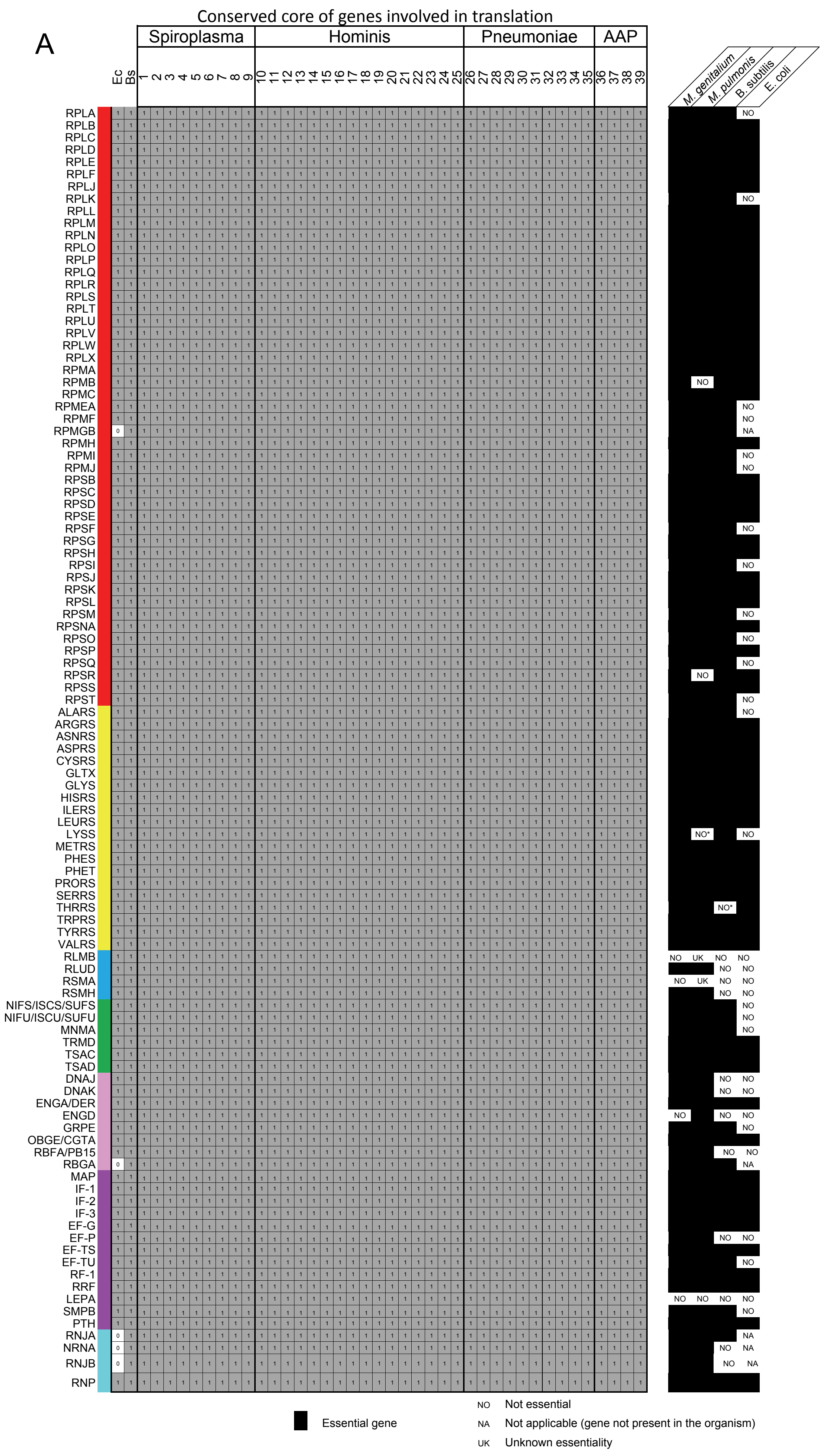
A
Conserved core of genes involved in translation
Spiroplasma
Hominis
Pneumoniae
AAP
Ec
Bs
M. genitalium
M. pulmonis
B. subtilis
E. coli
Essential gene
NO Not essential
NA Not applicable (gene not present in the organism)
UK Unknown essentiality

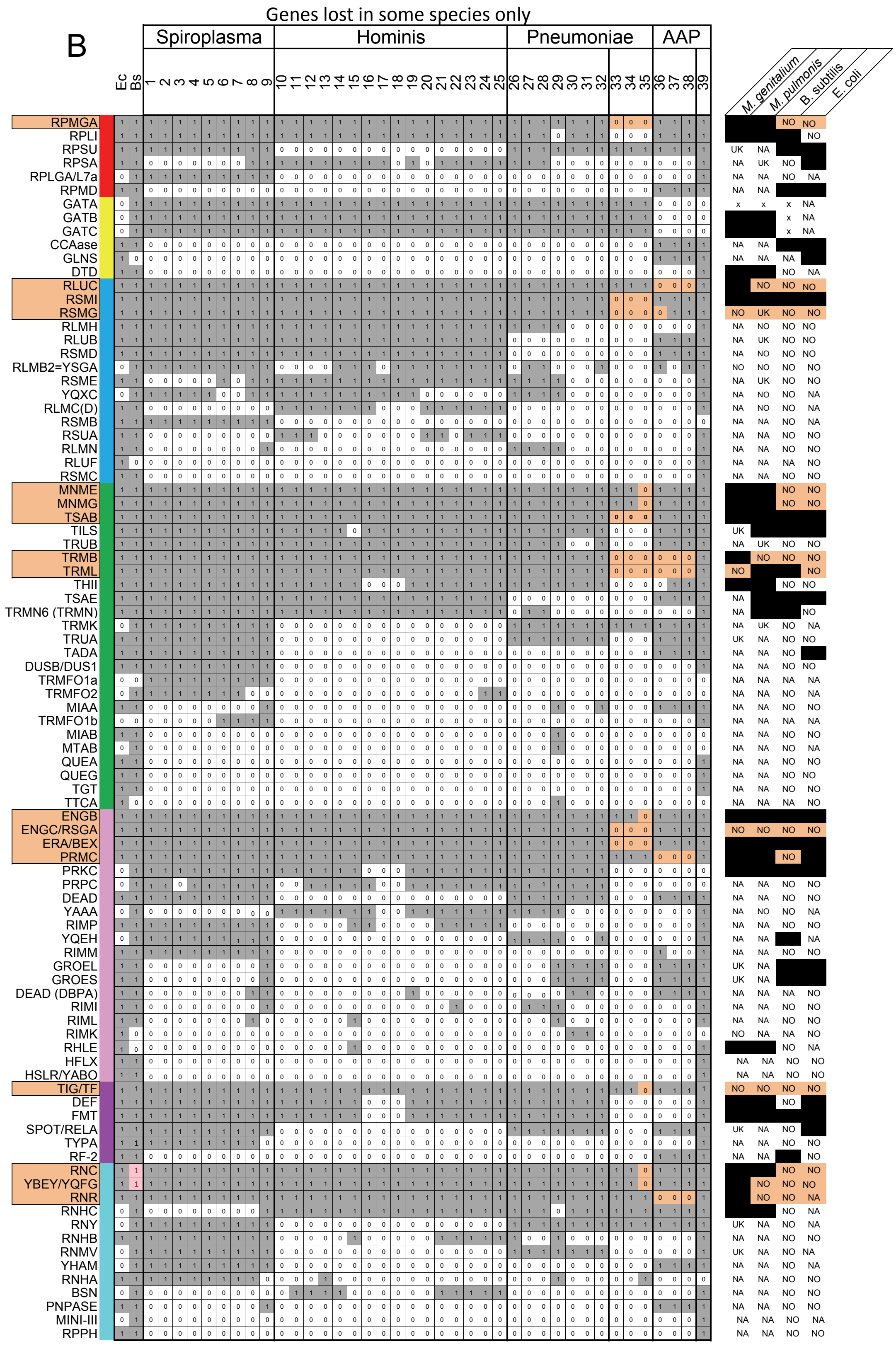

1 *M. capricolum* subsp. *capripneumoniae*
2 *M. leachii*
3 *M. mycoides* subsp. *mycoides*
4 *M. mycoides* subsp. *capri*
5 *M. capricolum* subsp. *capricolum*
6 *M. yeatsii*
7 *M. putrefaciens*
8 *Mesoplasma florum*
9 *Spiroplasma citri*
10 *M. auris*
11 *M. alkalescens*
12 *M. arginini*
13 *M. arthritidis*
14 *M. hominis*
15 *M. mobile*
16 *M. hyorhinis*
17 *M. ovipneumoniae*
18 *M. hyopneumoniae*
19 *M. pulmonis*
20 *M. synoviae*
21 *M. crocodyli*
22 *M. fermentans*
23 *M. bovigenitalium*
24 *M. bovis*
25 *M. agalactiae*
26 *U. diversum*
27 *U. parvum*
28 *U. urealyticum*
29 *M. penetrans*
30 *M. pneumoniae*
31 *M . genitalium*
32 *M. gallisepticum*
33 *M. haemofelis*
34 *M. haemocanis*
35 *M. suis*
36 *Phytoplasma mali*
37 *Phytoplasma australiense*
38 *Phytoplasma asteris*
39 *Acholeplasma laidlawii*
