# Supplementary material for: Predicting the Minimal Translation Apparatus: Lessons from the Reductive Evolution of Mollicutes
Source: PLoS Genet. 2014 May 8;10(5):e1004363. doi: 10.1371/journal.pgen.1004363 (PMC4014445; doi:10.1371/journal.pgen.1004363)
Supplement: Figure S3 — Dispensable proteins of translation apparatus in Bacteria. General information is the same as in Figure 4, except that results are now divided into 2 main boxes: part A corresponds to the proteins that are easily lost during reductive Mollicutes evolution and part B corresponds to proteins that have not been found in any of the 39 Mollicutes analyzed. Proteins are classified according to the 7 categories defined in Table 1. Acronyms indicated in black italics letters correspond to proteins absent in many Mollicutes but found in both E. coli and B. subtilis; in red italic letters are proteins absent in E. coli and present in B. subtilis, whereas proteins present in E. coli and absent in B. subtilis are indicated in bold green letters. Several DEAD-box helicases exist in these two bacteria, while none or a maximum two of these helicases are found in the different Mollicutes (see Table S3). All numbers in brackets within boxes correspond to those indicated in part D of Figure 1. (PDF) [file pgen.1004363.s003.pdf]

A. Easily lost proteins in Mollicutes

Ribosomal proteins (3)

S1 + *L7a* + *L30*

rRNA modification (11)

RsmB (m^5C_{967}) + *RsmD* (m^2G_{966})
RsmC (m^2G_{1207}) + *RsmE* (m^3U_{1498})
RsuA (Ψ_{516}) + *RluF* (Ψ_{2604}) + *RluB* (Ψ_{2605})
RlmCD ($m^5U_{747/1939}$) + *RlmH* ($m^3\Psi_{1915}$)
RlmN (dual-specific) m^2A_{2503}+m^2A_{37}/tRNA
RlmB2<or>**YqxC** (Gm_{2553}?)

Rib. assembly + prot maturation (16)

YqeH +*HflX* (GTPase) *30S/50S assembly*
RimP + *RimM* *30S maturases*
YaaA *Ribosome binding protein*
2 out of 4 *DBH's* + ***RhlE*** *Helicases*
GroEL + *GroES* + *HslR* *Protein folding*
RimI + *RimL* *S18/L7-acetyl-transferases*
RimK *S6-Glu-transferase*
PrkC + *PrpC* *Prot. (de)phosphorylation*

RNA processing (9)

BsN+*RNaseY*+*RNase M5* *Endonucleases*
PNPase+*Mini-III* + *YhaM* *Exonucleases*
RppH *5'-Pyrophosphohydrolase*
RNaseHI<or>*RNaseHII*<or>*RNaseHIII* = 2

tRNA modification (16)

In anticodon loop
TadA (I_{34})
QueA + *QueG* +*TgT* (Q_{34}-derivatives)
MiaA +*MiaB* ($ms^2i^6A_{37}$) + *TrmN* (m^6A_{37})
TsaE (subunit t^6A_{37}) + *MtaB* ($\underline{ms}^2t^6A_{37}$)
TruA ($\Psi_{38\text{-}39\text{-}40}$) + ***TtcA*** ($s^2C_{32}$)
Out of anticodon loop
DusB ($D_{16\text{-}17}$) + ThiI (s^4U_8)
TrmK ($m^1A^+_{22}$) + TruB (Ψ_{55})
TrmFO (m^5U_{54} *or targets unknown*)

tRNA aminoacylation (3 or 5)

GlnRS (<or> **GatA-GatB-GatC**)
DtDase *D-Tyr-deacylase*
CCAase (if CCA end is not encoded)

Translation factors (5)

FMT + *DEF* *tRNA de/formylation*
TypA (GTPase) *Ribosome association*
SpoT-RelA (fusion)=1 *ppGpp alarmone*
RF2 (if UGA codes for Stop)*Termination*

Total: 63

B. Absent in all Mollicutes

Ribosomal proteins (5)

S14b + ***S22*** + *L25* (stress protein) + L7b + L31

rRNA modification (14)

RsmF (m^5C_{1407}) + *RsmJ* (m^2G_{1516})
RlmA (m^1G_{745}) + *RlmI* (m^5C_{1962})
RlmD (m^5U_{747})
RlmF (m^6A_{1618}) + ***RlmG*** (m^2G_{1835})
RlmKL fusion prot.($m^7G^+_{2069}$+m^2G_{2545})
RlmJ (m^6A_{2030}) + ***RlmE*** (Um_{2552})
RluE (Ψ_{2457}) + ***RlmM*** (Cm_{2492})
RluA (dual-specific Ψ_{746}+ Ψ_{32}/tRNA)
YfjO (m^5U - target unknown)

Rib. assembly + prot maturation (10)

HfQ *Chaperones*
RimF *Rib. modulation factor*
YjgA + *YbhY*+***YibL*** *Rib. association prot*
PrmA *L11 methyl-transferase*
RimJ *S5 acetyl-transferase*
PrmB *L3 Glu-transferase*
RimO + ***YcaO*** *S12- ms2 transferase*

RNA processing (10)

RNase BN/Z +***RNase A***+***RNase E***+***RNase G*** *Endonucleases*
NrnB + ***Orn*** *Nano-exo-5'-nucleases*
RNase PH +***RNase T***+***RNase B***+***RNase D*** *Exonucleases*

tRNA modification (17)

In anticodon loop
CmoA + ***CmoB*** (cmo^5U_{34})
YadB ($\underline{Glu}Q_{34}$) +***TmcA*** (ac^4C_{34})
MnmC ($\underline{mnm}^5U_{34}$) + ***SelU*** (seU_{34})
TsaA ($\underline{m}^6t^6A_{37}$) + *TcdA* (ct^6A_{37})
TrmJ (Cm_{32}/Um_{32})
TusA + *(**TusB,C,D,E**)=1* (s^2U_{34})
Out of anticodon loop
DusA +***DusC*** ($D_{20\text{-}20a}$) +***TrmH*** (Gm_{18})
TrmA (m^5U_{54})+***TruD*** (Ψ_{13})+***TruC*** (Ψ_{65})
NifZ (S-relay)

tRNA aminoacylation (7)

GlyQ (α-subunit) +*ThrZ*+*TyrZ*+ ***LysU***
SelA + **SelD** Sec-$tRNA^{Sec}$ formation
YbaK editing subunit

Translation factors (5)

SelB requires **$tRNA^{Sec}_{UCA}$** *Elongation*
RF3 *Termination*
ArfA+***YaeJ***+*RsfA* *Rescue/silencing fact*

Total: 68
