# Supplementary material for: Predicting the Minimal Translation Apparatus: Lessons from the Reductive Evolution of Mollicutes
Source: PLoS Genet. 2014 May 8;10(5):e1004363. doi: 10.1371/journal.pgen.1004363 (PMC4014445; doi:10.1371/journal.pgen.1004363)
Supplement: Figure S4 — Genes coding for proteins implicated in translation in Mycoplasma genitalium, in addition to the core set of proteins. The data and symbols (species numbering, acronyms and their corresponding color codes on the left, meaning of grey background within the table) are the same as those of Figure 1, part B entitled ‘Genes lost in some Mollicutes species only’. Data concerning the M. genitalium (species # 31) are boxed with yellow. All acronyms in bold letters on the left correspond to proteins that are present in M. genitalium (see in Figure S3) and also present in other Mollicutes (orange background) or in contrary absent in other Mollicutes (light green background). The MPSM (Minimal Protein Synthesis Machinery) of Mollicutes includes all the 24 proteins encoded by genes in the orange background (only RlmB2/YqxC is missing in M. genitalium). The light green background of the other acronyms small boxes (16 cases) means that the corresponding proteins do not belong to the MPSM, but the protein are present in M. genitalium. (PDF) [file pgen.1004363.s004.pdf]

Proteins of *M. genitalium involved in translation,* in addition to the 104 ubiquitous core proteins

Function

Blue color means correlation with special properties of t-r- or mRNA

			Spiroplasma									Hominis																Pneumoniae										AAP				Function
	Ec	*Bs*	*1*	*2*	*3*	*4*	*5*	*6*	*7*	*8*	*9*	*10*	*11*	*12*	*13*	*14*	*15*	*16*	*17*	*18*	*19*	*20*	*21*	*22*	*23*	*24*	*25*	*26*	*27*	*28*	*29*	*30*	***31***	*32*	*33*	*34*	*35*	*36*	*37*	*38*	*39*	
**RPMGA**	1	1	1	1	1	1	1	1	1	1	1	1	1	1	1	1	1	1	1	1	1	1	1	1	1	1	1	1	1	1	1	1	1	1	0	0	0	1	1	1	1	**L33a r-Protein possibly belongs to distinct 50S as its paralogue L33b**
**RPLI**	1	1	1	1	1	1	1	1	1	1	1	1	1	1	1	1	1	1	1	1	1	1	1	1	1	1	1	1	1	1	0	1	1	1	0	0	0	1	1	1	1	**L9 r-Protein interacts with P-tRNA and limits ribosome frameshifting**
**RPSU**	1	1	1	1	1	1	1	1	1	1	1	0	0	0	0	0	0	0	0	0	0	0	0	0	0	0	0	1	1	1	1	1	1	1	1	1	1	1	1	1	1	**S21 r-Protein is involved in initiation step = important when S1 is absent**
RPSA	1	1	0	0	0	0	0	0	0	1	1	1	1	1	1	1	1	1	1	0	1	0	1	1	1	1	1	1	1	1	0	0	0	0	0	0	0	0	0	0	1	*<<< SI r-Protein dispensable when **leaderless mRNA** is initiated on 70S*
RPLGA/L7a	0	1	1	1	1	1	1	1	1	1	1	0	0	0	0	0	0	0	0	0	0	0	0	0	0	0	0	0	0	0	0	0	0	0	0	0	0	0	0	0	1	*<<< L7Ae (archaea-like) r-Protein present in B. subtilis, not in E. coli (gram-)*
RPMD	1	1	0	0	0	0	0	0	0	0	0	0	0	0	0	0	0	0	0	0	0	0	0	0	0	0	0	0	0	0	0	0	0	0	0	0	0	1	1	1	1	*< << L30 rProtein is absent in many bacteria other than Mollicutes*
**GATA**	0	1	1	1	1	1	1	1	1	1	1	1	1	1	1	1	1	1	1	1	1	1	1	1	1	1	1	1	1	1	1	1	1	1	1	1	1	0	0	0	0	**GatA+B+C is a multiprotein complex that allows amidation of Glu residue**
**GATB**	0	1	1	1	1	1	1	1	1	1	1	1	1	1	1	1	1	1	1	1	1	1	1	1	1	1	1	1	1	1	1	1	1	1	1	1	1	0	0	0	0	**on Glu-tRNA-Gln. All 3 subunits become essential when GlnRS is absent**
**GATC**	0	1	1	1	1	1	1	1	1	1	1	1	1	1	1	1	1	1	1	1	1	1	1	1	1	1	1	1	1	1	1	1	1	1	1	1	1	0	0	0	0	
GLNRS	1	1	0	0	0	0	0	0	0	0	0	0	0	0	0	0	0	0	0	0	0	0	0	0	0	0	0	0	0	0	0	0	0	0	0	0	0	1	1	1	1	*<<< Essential GlnRS is used only when GatA+B+C is absent*
CCAses	1	0	0	0	0	0	0	0	0	0	0	0	0	0	0	0	0	0	0	0	0	0	0	0	0	0	0	0	0	0	0	0	0	0	0	0	0	1	1	1	1	*<<< CCAse is dispensable ONLY when **3'CCA-tail of pre-tRNAs** is encoded*
DTD	1	1	0	0	0	0	0	0	0	0	0	0	0	0	0	0	0	0	0	0	0	0	0	0	0	0	0	0	0	0	0	0	0	0	0	0	0	0	0	0	1	*<<< catalyzes deacylation of mischarged D-Tyr-tRNA-Tyr (editing function)*
**RLUC**	1	1	1	1	1	1	1	1	1	1	1	1	1	1	1	1	1	1	1	1	1	1	1	1	1	1	1	1	1	1	1	1	1	1	1	1	1	0	0	0	1	**catalyzes essential Ψ's (2504+2580) in PTCenter (h18) of 23S rRNA**
**RSMI**	1	1	1	1	1	1	1	1	1	1	1	1	1	1	1	1	1	1	1	1	1	1	1	1	1	1	1	1	1	1	1	1	1	1	0	0	0	1	1	1	1	**catalyzes essential Cm1402 in helix 44 of 16S rRNA in decoding site**
**RSMG**	1	1	1	1	1	1	1	1	1	1	1	1	1	1	1	1	1	1	1	1	1	1	1	1	1	1	1	1	1	1	1	1	1	1	0	0	0	0	1	1	1	**catalyzes formation of a positively charged m7G527 in h18 of 16S rRNA**
RLMH	1	1	1	1	1	1	1	1	1	1	1	1	1	1	1	1	1	1	1	1	1	1	1	1	1	1	1	1	1	1	1	0	0	0	0	0	0	0	0	0	1	*<<< persistent methylase, late formation of m3 Ψ1915 in H69 within 70S*
RLUB	1	1	1	1	1	1	1	1	1	1	1	1	1	1	1	1	1	1	1	1	1	1	1	1	1	1	1	0	0	0	0	0	0	0	0	0	0	1	1	1	1	*<<< catalyzes Ψ2605 formation in helix 93 ot the PTCenter of 23S rRNA*
RSMD	1	1	1	1	1	1	1	1	1	1	1	1	1	1	1	1	1	1	1	1	1	1	1	1	1	1	1	0	0	0	0	0	0	0	0	0	0	1	1	1	1	*<<< catalyzes m2G966 in helix 31 of decoding center of the ribosome*
RLMB2=YSGA	0	1	1	1	1	1	1	1	1	1	1	0	0	0	0	1	1	1	0	1	1	1	1	1	1	1	1	0	1	1	0	0	0	1	0	0	0	1	0	1	1	*<<< RlmB2 (or YqxC) probably catalyzes the essential Gm2553 in the A-loop*
YQXC	0	1	1	1	1	1	1	0	0	1	1	1	1	1	1	1	1	1	1	1	1	0	0	0	0	0	0	1	1	1	1	0	0	0	0	0	0	0	0	0	1	*<<< YqxC (or RlmB2) probably catalyzes Gm2553 in the A-loopof PTCenter*
RSME	1	1	0	0	0	0	0	1	0	1	1	1	1	1	1	1	1	1	1	1	1	1	1	1	1	1	1	1	1	1	1	0	0	0	0	0	0	0	0	0	1	*<<< catalyzes m3U1498 in helix 44 of the decoding center of the ribosome*
RLMC(D)	1	1	0	0	0	0	0	0	0	0	0	1	1	1	1	1	1	1	0	0	0	1	1	1	1	1	1	0	0	0	0	0	0	0	0	0	0	0	0	0	1	*<< dual specific m5U rRNA Mtase acting at U1939 and/or U749 in 23S rRNA*
RSMB	1	1	1	1	1	1	1	1	1	1	1	0	0	0	0	0	0	0	0	0	0	0	0	0	0	0	0	0	0	0	0	0	0	0	0	0	0	0	0	0	0	*<< catalyzes m5C967, adjacent to m2G966(RsmD) in h31 of decoding Center*
RSUA	1	1	0	0	0	0	0	0	0	0	0	1	1	1	0	0	0	0	0	0	0	1	1	0	1	1	1	0	0	0	0	0	0	0	0	0	0	0	0	0	1	*<<< catalyzes Ψ516 in helix 18 of 30S subunit*
RLMN	1	1	0	0	0	0	0	0	0	0	1	0	0	0	0	0	0	0	0	0	0	0	0	0	0	0	0	1	1	1	1	0	0	0	0	0	0	0	0	0	1	*<<< in E. coli this enz. is dual-specific for m2A2503 in rRNA & m2A37 in tRNA*
RLUF	1	0	0	0	0	0	0	0	0	0	0	0	0	0	0	0	0	0	0	0	0	0	0	0	0	0	0	0	0	0	0	0	0	0	0	0	0	0	0	0	1	*<<< catalyzes Ψ2604, it is present in gram negative bacteria only*
RSMC	1	1	0	0	0	0	0	0	0	0	0	0	0	0	0	0	0	0	0	0	0	0	0	0	0	0	0	0	0	0	0	0	0	0	0	0	0	0	0	0	1	*<< catalyzes m2G1907, this enzyme has been lost very early during evolution*
**MNME**	1	1	1	1	1	1	1	1	1	1	1	1	1	1	1	1	1	1	1	1	1	1	1	1	1	1	1	1	1	1	1	1	1	1	1	1	0	1	1	1	1	**MnmE + MnmG form a complex that catalyzes cmnm5U34 formation in**
**MNMG**	1	1	1	1	1	1	1	1	1	1	1	1	1	1	1	1	1	1	1	1	1	1	1	1	1	1	1	1	1	1	1	1	1	1	1	1	0	1	1	1	1	**tRNA.This wobble base modification allows decoding split(2)-codon boxes**
**TSAB**	1	1	1	1	1	1	1	1	1	1	1	1	1	1	1	1	1	1	1	1	1	1	1	1	1	1	1	1	1	1	1	1	1	1	0	0	0	1	1	1	1	**Together with TsaC+TsaD = essential complex for t6A37 formation in tRNA**
**TILS**	1	1	1	1	1	1	1	1	1	1	1	1	1	1	1	1	0	1	1	1	1	1	1	1	1	1	1	1	1	1	1	1	1	1	0	0	0	1	1	1	1	**lack of TilS requires a C-to-U34 mutation in tRNAIle (CAU)**
TRUB	1	1	1	1	1	1	1	1	1	1	1	1	1	1	1	1	1	1	1	1	1	1	1	1	1	1	1	1	1	1	1	0	0	1	0	0	0	1	1	1	1	*<<< a persistant enzyme for Ψ55 formation in T Ψ-loop of tRNA*
**TRMB**	1	1	1	1	1	1	1	1	1	1	1	1	1	1	1	1	1	1	1	1	1	1	1	1	1	1	1	1	1	1	1	1	1	1	0	0	0	0	0	0	1	**catalyses formation of a positively charged m7G46 in tRNA**
**TRML**	1	1	1	1	1	1	1	1	1	1	1	1	1	1	1	1	1	1	1	1	1	1	1	1	1	1	1	1	1	1	1	1	1	1	0	0	0	0	0	0	1	**essential for Um34 formation in tRNA and decoding split(2)-codon boxes**
THII	1	1	1	1	1	1	1	1	1	1	1	1	1	1	1	1	1	0	0	0	1	1	1	1	1	1	1	1	1	1	1	1	1	1	0	0	0	0	1	1	1	*<<< this enzyme catalyzes S4U8 in tRNA, requires IcsS<and/or>SufU*
TSAE	1	1	1	1	1	1	1	1	1	1	1	1	1	1	1	1	1	1	1	1	1	1	1	1	1	1	1	0	0	0	0	0	0	0	0	0	0	1	1	1	1	*<<< together with TsaB+TsaC+TsaD, it facilitates t6A37 formation*
TRMN6 (TRMN)	1	1	1	1	1	1	1	1	1	1	1	1	1	1	1	1	1	1	1	1	1	1	1	1	1	1	1	0	1	1	0	0	0	0	0	0	0	0	0	0	1	*<<< catalyzes formation of m6A37 in anticodon loop of tRNA*
TRMK	0	1	1	1	1	1	1	1	1	1	1	0	0	0	0	0	0	0	0	0	0	0	0	0	0	0	0	1	1	1	1	1	1	1	1	1	1	1	1	1	1	*<<< catalyzes formation of positively charged m1A22 in D-loop of tRNA*
TRUA	1	1	1	1	1	1	1	1	1	1	1	0	0	0	0	0	0	0	0	0	0	0	0	0	0	0	0	1	1	1	1	1	1	1	0	0	0	1	1	1	1	*<<< catalyzes formation of Ψ38-39-40 in anticodon loop of tRNA*
TADA	1	1	1	1	1	1	1	1	1	1	1	0	0	0	0	0	0	0	0	0	0	0	0	0	0	0	0	0	0	0	0	0	0	0	0	0	0	1	1	1	1	*<<< TadA (I34 in tRNA) can be lost if **A-to-U mutation occurs in tRNA-Arg***
DUSB/DUS1	1	1	1	1	1	1	1	1	1	1	1	0	0	0	0	0	0	0	0	0	0	0	0	0	0	0	0	0	0	0	0	0	0	0	0	0	0	0	0	0	1	*<<< catalyzes dihydrouridine formation in the D-loop of tRNA*
TRMFO1a	0	0	1	1	1	1	1	1	1	1	1	0	0	0	0	0	0	0	0	0	0	0	0	0	0	0	0	0	0	0	0	0	0	0	0	0	0	0	0	0	0	*<<< putative RNA:m5U folate-dependent MTase (target unknown)*
TRMFO1b	0	0	0	0	0	0	0	1	1	1	1	0	0	0	0	0	0	0	0	0	0	0	0	0	0	0	0	0	0	0	0	0	0	0	0	0	0	0	0	0	1	*<<< putative tRNA m5U54 folate-dependent methyltransferase*
TRMFO2	0	1	1	1	1	1	1	1	1	0	0	0	0	0	0	0	0	0	0	0	0	0	0	0	0	1	1	0	0	0	0	0	0	0	0	0	0	0	0	0	0	*<<< putative RNA:m5U folate-dependent Mtase (target unknown)*
MIAA	1	1	0	0	0	0	0	0	0	0	1	0	0	0	0	0	0	0	0	0	0	0	0	0	0	0	0	0	0	0	1	0	0	1	0	0	0	1	1	1	1	*<<< catalyses i6A37 formation in tRNA harboring A36 in anticodon*
MIAB	1	1	0	0	0	0	0	0	0	0	0	0	0	0	0	0	0	0	0	0	0	0	0	0	0	0	0	0	0	0	1	0	0	0	0	0	0	0	0	0	0	*<<< further hypermethylates i6A-containig tRNA into ms2i6A-tRNA*
MTAB	0	1	0	0	0	0	0	0	0	0	0	0	0	0	0	0	0	0	0	0	0	0	0	0	0	0	0	0	0	0	1	0	0	0	0	0	0	0	0	0	0	*<<< catalyses addition of ms2 group on t6A-containing tRNA (ms2t6A)*
TGT	1	1	0	0	0	0	0	0	0	0	0	0	0	0	0	0	0	0	0	0	0	0	0	0	0	0	0	0	0	0	0	0	0	0	0	0	0	0	0	0	1	*<<< tgt is a G-to-Q transglycosylase that works in concert with*
QUEA	1	1	0	0	0	0	0	0	0	0	0	0	0	0	0	0	0	0	0	0	0	0	0	0	0	0	0	0	0	0	0	0	0	0	0	0	0	0	0	0	1	*QueA and QueG to form various derivatives of the Q34 metabolism in*
QUEG	1	1	0	0	0	0	0	0	0	0	0	0	0	0	0	0	0	0	0	0	0	0	0	0	0	0	0	0	0	0	0	0	0	0	0	0	0	0	0	0	1	*a subset of tRNAs harbouring a central U35 in anticodon*
TTCA	1	0	0	0	0	0	0	0	0	0	0	0	0	0	0	0	0	0	0	0	0	0	0	0	0	0	0	0	0	0	1	0	0	0	0	0	0	0	0	0	0	*>> catalyses formation of s2C32 in tRNA (aroses after lateral gene transfer)*
**ENGB**	1	1	1	1	1	1	1	1	1	1	1	1	1	1	1	1	1	1	1	1	1	1	1	1	1	1	1	1	1	1	1	1	1	1	1	1	0	1	1	1	1	**essential GTPase allowing 50S subunit assembly/maturation**
**ENGC/RSGA**	1	1	1	1	1	1	1	1	1	1	1	1	1	1	1	1	1	1	1	1	1	1	1	1	1	1	1	1	1	1	1	1	1	1	0	0	0	1	1	1	1	**GTPase allowing 30S subunit assembly/maturation**
**ERA/BEX**	1	1	1	1	1	1	1	1	1	1	1	1	1	1	1	1	1	1	1	1	1	1	1	1	1	1	1	1	1	1	1	1	1	1	0	0	0	1	1	1	1	**essential GTPase allowing 30S subunit assembly/maturation**
**PRMC**	1	1	1	1	1	1	1	1	1	1	1	1	1	1	1	1	1	1	1	1	1	1	1	1	1	1	1	1	1	1	1	1	1	1	1	1	1	0	0	0	1	**essential methyltransferase for methylating RF-1 factor**
PRKC	0	1	1	1	1	1	1	1	1	1	1	1	1	1	1	1	1	0	0	0	1	1	1	1	1	1	1	1	1	1	1	1	1	1	0	0	0	0	0	0	0	*<<< PRKC/PrpC are protein kinase/phosphatase acting on EngC, EF-Tu and*
PRPC	0	1	1	1	0	1	1	1	1	1	1	0	0	1	1	1	1	1	0	0	1	1	1	1	1	1	1	1	1	1	1	1	1	1	0	0	0	0	0	0	0	*probably also on other proteins (regulatory purpose)*
DEAD	1	1	1	1	1	1	1	1	1	1	1	0	0	0	0	0	0	0	0	0	0	0	0	0	0	0	0	1	1	1	1	1	1	1	0	0	0	1	1	1	1	*<<< at least 1 of the 4 DEAD helicases in B. subtilis (5 in E. coli). Because*
DEAD (DBPA)	1	1	0	0	0	0	0	0	0	1	1	0	0	0	0	0	0	0	0	0	1	0	0	0	0	0	0	0	0	0	0	1	1	0	0	0	0	1	1	1	1	*Mollicutes **RNAs are A/T rich** , several helicases probably become obsolete*
RHLE	1	0	0	0	0	0	0	0	0	0	0	0	0	0	0	0	1	0	0	0	0	0	0	0	0	0	0	0	0	0	0	0	0	0	0	0	0	0	0	0	1	*<<< Homolog of 1 of the 5 helicases in E. coli, probably arose by LGT*
YAAA	0	1	0	0	0	0	0	0	0	0	0	1	1	1	1	1	1	1	0	0	1	1	1	1	1	1	1	1	1	1	1	0	0	0	0	0	0	0	0	0	1	*<<< YaaA is a ribosome binding protein involved in ribosome maturation*
RIMP	1	1	1	1	1	1	1	1	1	1	1	0	0	0	0	0	1	1	0	0	0	0	1	1	1	1	1	0	0	0	0	0	0	0	0	0	0	0	0	0	1	*<<< RimP and RimM are 2 pre-ribosome binding proteins acting before*
RIMM	1	1	1	1	1	1	1	1	1	1	1	0	0	0	0	0	0	0	0	0	0	0	0	0	0	0	0	0	0	0	0	0	0	0	0	0	0	1	0	0	1	*2 other (non GTPase) RbfA/EngC at late steps of 30S subunit assembly*
YQEH	0	1	1	1	1	1	1	1	1	1	1	0	0	0	0	0	0	0	0	0	0	0	0	0	0	0	0	1	1	1	1	0	0	1	0	0	0	0	0	0	1	*<<< GTPase of gram + bacteria, acting at early step of 30S maturation*
GROEL	1	1	0	0	0	0	0	0	0	0	1	0	0	0	0	0	0	0	0	0	0	0	0	0	0	0	0	0	0	0	1	1	1	1	0	0	0	1	1	1	1	*<<< non persistant GroEL/GroES belonging to the polypetide chaperone*
GROES	1	1	0	0	0	0	0	0	0	0	1	0	0	0	0	0	0	0	0	0	0	0	0	0	0	0	0	0	0	0	1	1	1	1	0	0	0	1	1	1	1	*networking together with the essential DnaK/DnaJ/GrpE and Tig*
RIMI	1	1	0	0	0	0	0	0	0	0	1	0	0	0	0	0	0	0	0	0	0	0	0	1	0	0	0	0	1	1	1	0	0	0	0	0	0	0	0	0	1	*<<< Both RimI and RimL catalyze posttranslational acetylation of r-proteins,*
RIML	1	1	0	0	0	0	0	0	0	1	0	0	0	0	0	0	1	0	0	0	0	0	0	0	0	0	0	0	0	0	1	0	0	0	0	0	0	0	0	0	1	*S18 and L12 respectively; the acetylated form of L12 being designated L7*
RIMK	1	0	0	0	0	0	0	0	0	0	0	0	0	0	0	0	0	0	0	0	0	0	0	0	0	0	0	0	0	0	0	1	1	0	0	0	0	0	0	0	0	*<<< RimK catalyzes addition of glutamic acid residue to C-ter of S6 r-Protein*
HFLX	1	1	0	0	0	0	0	0	0	0	0	0	0	0	0	0	0	0	0	0	0	0	0	0	0	0	0	0	0	0	0	0	0	0	0	0	0	0	0	0	1	*<<< GTPase of the ObgE/CtgA superfamily, obviously obsolete in Mollicutes*
HSLR/YABO	1	1	0	0	0	0	0	0	0	0	0	0	0	0	0	0	0	0	0	0	0	0	0	0	0	0	0	0	0	0	0	0	0	0	0	0	0	0	0	0	1	*<<< chaperone named Hsp15, a ribosome-associated heat-shock protein*
**TIG/TF**	1	1	1	1	1	1	1	1	1	1	1	1	1	1	1	1	1	1	1	1	1	1	1	1	1	1	1	1	1	1	1	1	1	1	1	1	0	1	1	1	1	**essential Isomerase and ribosomal chaperone for folding nascent proteins**
DEF	1	1	1	1	1	1	1	1	1	1	1	1	1	1	1	1	1	0	0	0	1	1	1	1	1	1	1	1	1	1	1	1	1	1	0	0	0	0	0	0	1	*<< FMT and DEF, catalyzing tRNA formylation & deformylation respectively*
FMT	1	1	1	1	1	1	1	1	1	1	1	1	1	1	1	1	1	0	0	0	1	1	1	1	1	1	1	1	1	1	1	1	1	1	0	0	0	0	0	0	1	*FMT and DEF always disapear together*
SPOT/RELA	1	1	1	1	1	1	1	1	1	1	1	0	0	0	0	0	0	0	0	0	0	0	0	0	0	0	0	1	1	1	1	1	1	1	0	0	0	1	1	1	1	*<<< fused enyme activities into 1 protein 'alarmone' for Stress response*
TYPA	1	1	1	1	1	1	1	1	1	1	0	0	0	0	0	0	0	0	0	0	0	0	0	0	0	0	0	0	0	0	0	0	0	0	0	0	0	0	0	0	1	*<<< GTPase playing a translation regulatory role during stress-related events*
RF-2	1	1	0	0	0	0	0	0	0	0	0	0	0	0	0	0	0	0	0	0	0	0	0	0	0	0	0	0	0	0	0	0	0	0	0	0	0	1	1	1	1	*<<< lack of RF-2 correlates with the emergence of a special tRNATrpU*CA*
**RNC**	1	1	1	1	1	1	1	1	1	1	1	1	1	1	1	1	1	1	1	1	1	1	1	1	1	1	1	1	1	1	1	1	1	1	1	1	0	1	1	1	1	**essensial single-strand endonuclease RNase III**
**YBEY/YQFG**	1	1	1	1	1	1	1	1	1	1	1	1	1	1	1	1	1	1	1	1	1	1	1	1	1	1	1	1	1	1	1	1	1	1	1	1	0	1	1	1	1	**essential 3'-5' exonuclease - specificity overlap with those of RNR/PNPase**
**RNR**	1	1	1	1	1	1	1	1	1	1	1	1	1	1	1	1	1	1	1	1	1	1	1	1	1	1	1	1	1	1	1	1	1	1	1	1	1	0	0	0	1	**lack of RNR could probably be complemented by PNPase and/or YhaM**
PNPASE	1	1	0	0	0	0	0	0	0	0	1	0	0	0	0	0	0	0	0	0	0	0	0	0	0	0	0	0	0	0	0	0	0	0	0	0	0	1	1	1	1	*<<< lack of PNPase could probably be complemented by RNR (3'-5'-exonucl.)*
YHAM	0	1	1	1	1	1	1	1	1	1	1	0	0	0	0	0	0	0	0	0	0	0	0	0	0	0	0	0	0	0	0	0	0	0	0	0	0	1	1	1	1	*<<< 3'-5' exonucl. activity as YbeY, RNR, PNPase (overlapping specificity)*
RNHB	1	1	1	1	1	1	1	1	1	1	1	0	0	0	0	0	1	0	0	0	0	0	1	1	1	1	1	1	0	0	1	0	0	0	0	0	0	0	0	0	1	*<<< RNases HI, HII and HIII are multivariants present in all bacteria,*
RNHA	1	1	1	1	1	1	1	1	1	1	0	0	0	0	1	0	0	0	0	0	0	0	0	0	0	0	0	0	0	0	1	0	0	0	0	0	1	0	0	0	0	*<<< they clive RNA of RNA-DNA hybrids to prevent aberrant DNA replication*
**RNHC**	0	1	0	0	0	0	0	0	0	0	1	1	1	1	1	1	1	1	1	1	1	1	1	1	1	1	1	1	1	1	0	1	1	1	1	1	1	1	1	1	1	**at sites other than oriC, only 1 or 2 variant(s) is (are) present**
RNY	0	1	1	1	1	1	1	1	1	1	1	0	0	0	0	0	0	0	0	0	0	0	0	0	0	0	0	1	1	1	1	1	1	1	1	1	1	1	1	1	1	*<<< Endoribonuclease that, together with RnmV (M5), PNPase and the*
RNM5	0	1	1	1	1	1	1	1	1	1	1	0	0	0	0	0	0	0	0	0	0	0	0	0	0	0	0	1	1	1	1	1	1	1	0	0	0	0	0	0	1	*essential RNase J1+RNase J2, are normally involved in mRNA degradation*
BSN	0	1	0	0	0	0	0	0	0	0	0	0	1	1	1	1	0	0	0	0	0	0	1	1	1	1	1	0	0	0	0	0	0	0	0	0	0	0	0	0	1	*<<< RNase typical of B. subtilis, the function ins Mollicute is not clear*
MINI-III	0	1	0	0	0	0	0	0	0	0	0	0	0	0	0	0	0	0	0	0	0	0	0	0	0	0	0	0	0	0	0	0	0	0	0	0	0	0	0	0	1	*<<< Bifunctional oligoribonuclease & PAP phosphatase, works as RNase J2*
RPPH	1	1	0	0	0	0	0	0	0	0	0	0	0	0	0	0	0	0	0	0	0	0	0	0	0	0	0	0	0	0	0	0	0	0	0	0	0	0	0	0	1	*<<< Pyrophophohydrolase, perform similar job the essential RNase J1*
