# Supplementary material for: Predicting the Minimal Translation Apparatus: Lessons from the Reductive Evolution of Mollicutes
Source: PLoS Genet. 2014 May 8;10(5):e1004363. doi: 10.1371/journal.pgen.1004363 (PMC4014445; doi:10.1371/journal.pgen.1004363)
Supplement: Figure S5 — Protein synthesis machinery in bacteria with reduced genomes. On the left part of the figure are listed the acronyms of the 129 proteins of the MPSM (Minimal Protein Synthesis Machinery) deduced from the comparison of genomes of 39 Mollicutes (see Figure 4 and corresponding text in the main part of the manuscript). The central part of the figure is the common set of 111 proteins involved in the ribosome biogenesis and mRNA translation of 5 obligate bacterial endosymbionts of insects (Buchnera aphidicola strains BBp, Bap, BSg/618, 652, 653 Kbp respectively, Candidatus Blochmannia floridanus/710 Kbp and Wigglesworthia glossinidia/700 Kbp) and listed in Table 1 of the paper by Gil, Silva, Pereto and Moya [139]. On the left part of the figure is the common set of 97 translation proteins in 2 obligate insect symbionts (Sulcia Muelleri/190 Kbp and Nasuia deltocephlinicola/112 Kbp) cohabiting the same host cell Macrosteles quadrilineatus), listed in tables 2 and 3 of supplemental materials of the paper published by Bennett and Moran [138]. The acronyms and corresponding color code for the boxes are as in Table 1 and Figure 1 of the main text, the corresponding names being given in Table S2. Acronyms indicated in black bold letters correspond to proteins present in E. coli and B. subtilis, in bold red letters to proteins found in B. subtilis and not in E. coli, and in bold Green letters to proteins found in Nasuia only, not in the co-symbiont Sulcia. All numbers in brackets correspond to the total proteins found in each of the protein family boxes. The purpose of this comparison is to point out that the translation proteins identified as highly resistant to genomic erosion during Mollicutes evolution are the ones that are also resistant to genomic erosion in Insect endosymbionts. Moreover, when two obligate endosymbionts co-exist in the same host cell, some important proteins exist in only one of the two endosymbionts, attesting for probable functional complementation. A ma [file pgen.1004363.s005.pdf]

Minimal protein synthesis machinery in bacteria with reduced genomes

	in 39 Mollicutes (MPSM)	in 5 Endosymbionts	in 2 co-Endosymbionts
**Ribosomal proteins**	(52) **S2 to S20** + **S21**(<or>S1) **L1 to L36** *(lack L7a,b, L25, L30, L31b, L33a)*	(51) **Same as in Mollicutes** *(except lack S1 + S21)*	(52) **Almost as in Mollicutes** +**S1** + **L7** + **L25** *(Lack only L24, L26, L29, L30)* while **L15** in Nasuia
**rRNA modification**	(8) **RsmA** + **RsmG** + **RsmH** **RsmI** + **RluD** + **RluC** **RlmB** + **RlmB2**<or>**YqxC**=1	(3) **RsmA** + **RsmH** + **RsmI**	(5) Only **RsmA** + **RsmH** + **RsmI** +**RluD**, while **RsmD** in Nasuia
**Ribosome assembly and protein maturation**	(12) **RbfA** + **EngA** + **RbgA** **EngD** + **EngB** + **EngC** **ObgE** + **Era** + **PrmC** **DnaK** + **DnaJ** + **GrpE**	(12) **Almost as in Mollicutes** *(lack RbgA , EngB, EngC)* but + **GroEL** + **GroES** + **DeaD**	(9) **RbfA** + **EngA** + **ObgE** **DnaK** + **DnaJ** + **GrpE** also + **GroEL** + **GroES** while **PrmC** in Nasuia only
**RNA processing**	(8) **C5p(RnP)** + **YbeY** **n-RNase** (1<or>5)=1 **RNase J1** + **RNase J2** **RNase III** + **RNase R** **RNaseHI**<or>**HII**<or>**HIII**=*1*	(4) Only **C5p(RnP)** + **YbeY** + **RNase III** and + **PNPase**	(2) Only **C5p(RnP)** + **YbeY**
**tRNA modification**	(12) **TrmD** + **TrmL** + **TrmB** **TsaC** + **TsaD** + **TsaB** **TilS** <or>*$tRNA^{Ile}$(UAU)* **MnmA** + **MnmE** + **MnmG** **IcsS/U/NifS** + **SufU/S/NifU**= 2	(7) **Almost as in Mollicutes** *(lack TrmD, TsaC, TsaB) (no information on SufU/sufS/NifU)*	(10) Only **TilS** +**MnmA** +**MnmE** + **MnmG** +**TrmL** +**IcsS** +**SufS** but + **MiaA** + **MiaB** + **TruA**
**tRNA aminoacylation**	(23) **19+1 AA-tRNA synthetases** **+GatA+B+C** (<or> **GlnRS**) *(PheRS is heteromeric)*	(21) **19+1 AA-tRNA synthetases** **+ GlnRS** *(no GatA+B+C)* *(PheRS is heteromeric)*	(10) Only **7 AA-tRNA synth.** for Val,Gln,Tyr,Leu,Ser,Trp, Asn here **Ccase** is present while **GatA+GatB** in Nasuia,
**Translation factors**	(14) **IF-1** + **IF-2** + **IF-3** **EF-Tu+EF-Ts** +**EF-G** + **EF-P** **LepA** + **RF-1** + **MAP** + **PTH** **RRF** + **SmpB** + **Tig**	(13) **Same as in Mollicutes** *(except that Tig is not found)*	(11) Only **IF-1** +**IF-2** +**IF-3** + **DEF** **EF-Tu** +**EF-G** +**RF-1** +**SmpB** while **RRF** + **RF2** + **FMT** in Nasuia only
	**Total: 129 proteins**	**Total: 111 proteins**	**Total: 99 proteins**
