# Supplementary material for: Predicting the Minimal Translation Apparatus: Lessons from the Reductive Evolution of Mollicutes
Source: PLoS Genet. 2014 May 8;10(5):e1004363. doi: 10.1371/journal.pgen.1004363 (PMC4014445; doi:10.1371/journal.pgen.1004363)
Supplement: Table S1 — List of selected (39) Mollicutes with some genomics and phenotypic (cultivability) features. The list of the 39 selected Mollicutes is given following the numbering used throughout the manuscript. The genomics features (Genome size, % G+C, #CDS) were obtained from Genbank. The data from the six non-cultivated Mollicutes are framed with a red dashed line. (PDF) [file pgen.1004363.s006.pdf]

Table S1: List of selected mollicutes with some genomics and phenotypic (cultivability) features

Phylogenetic group	Numbering in this study	Mycoplasma species	Strain	Natural Host	Genome size (Mbp)	% G+C	# CDS	Cultivability	GenBank #
Spiroplasma	1	*M. capricolum* subsp. *capripneumoniae*	99108-P1	Ruminant	1.006	23.6	693	Yes	PRJNA164785
	2	*M. leachii*	PG50	Ruminant	1.009	23.7	883	Yes	CP002108.1
	3	*M. mycoides* subsp. *mycoides*	PG1	Ruminant	1.212	24.0	917	Yes	BX293980.2
	4	*M. mycoides* subsp. *capri*	95010	Ruminant	1.154	23.8	921	Yes	FQ377874.1
	5	*M. capricolum* subsp. *capricolum*	ATCC27343	Ruminant	1.010	23.8	817	Yes	CP000123.1
	6	*M. yeatsii*	13926	Ruminant	0.897	25.8	687	Yes	AORK00000000
	7	*M. putrefaciens*	CIRAD	Ruminant	0.860	27.0	684	Yes	CP004357.1
	8	*Mesoplasma florum*	L1	Plants	0.793	27.0	683	Yes	AE017263.1
	9	*Spiroplasma citri*	GII-3	Insects&plants	1.840	26.0	2050	Yes	PRJNA13930
Hominis	10	*M. auris*	15026	Ruminant	0.768	27.2	616	Yes	AORI00000000
	11	*M. alkalescens*	14918	Ruminant	0.772	25.6	601	Yes	AMWK00000000
	12	*M. arginini*	7264	Ruminant	0.616	26.2	513	Yes	AORG00000000
	13	*M. arthritidis*	158L3-1	Rodent	0.820	30.7	631	Yes	CP001047.1
	14	*M. hominis*	PG21	Human	0.665	27.1	531	Yes	FP236530.1
	15	*M. mobile*	163K	Fish	0.777	25.0	637	Yes	AE017308.1
	16	*M. hyorhinis*	HUB-1	Pig	0.840	25.9	663	Yes	CP002170.1
	17	*M. ovipneumoniae*	14811	Ruminant	1.071	29.2	685	Yes	PRJNA164759
	18	*M. hyopneumoniae*	232	Pig	0.893	28.6	695	Yes	AE017332.1
	19	*M. pulmonis*	UAB CTIP	Rodent	0.964	26.6	783	Yes	AL445566.1
	20	*M. synoviae*	53	Poultry	0.799	28.5	675	Yes	AE017245.1
	21	*M. crocodyli*	MP145	Crocodilians	0.934	26.9	697	Yes	CP001991.1
	22	*M. fermentans*	JER	Human	0.977	26.9	799	Yes	CP001995.1
	23	*M. bovigenitalium*	cl-51080	Ruminant	0.862	29.0	677	Yes	AORH00000000
	24	*M. bovis*	PG45	Ruminant	1.003	29.3	766	Yes	CP002188.1
	25	*M. agalactiae*	PG2	Ruminant	0.877	29.7	714	Yes	CU179680.1
Pneumoniae	26	*Ureaplasma diversum*	246	Ruminant	1.011	28.1	675	Yes	PRJNA164765
	27	*U. parvum*	ATCC700970	Human	0.752	25.5	614	Yes	AF222894.1
	28	*U. urealyticum*	ATCC 33699	Human	0.874	25.8	647	Yes	CP001184.1
	29	*M. penetrans*	HF2	Human	1.359	25.7	1040	Yes	BA000026.2
	30	*M. pneumoniae*	M129	Human	0.816	40.0	689	Yes	U00089.2
	31	*M . genitalium*	G37	Human	0.580	32.0	482	Yes	L43967.2
	32	*M. gallisepticum*	R (Low)	Poultry	0.996	31.4	727	Yes	AE015450.2
	33	*M. haemofelis*	Langford	Cat	1.147	38.8	1545	No	FR773153.2
	34	*M. haemocanis*	Illinois	Dog	0.920	35.3	1156	No	CP003199.1
	35	*M. suis*	KI3806	Pig	0.709	31.1	799	No	FQ790233.1
AAP	36	*Candidatus* Phytoplasma mali	AT	Insect&plant	0.602	21.4	480	No	CU469464.1
	37	*Candidatus* Phytoplasma australiense	rp-A	Insect&plant	0.880	27.4	844	No	AM422018.1
	38	*Candidatus* Phytoplasma asteris	Yellows	Insect&plant	0.707	26.8	694	No	AP006628.2
	39	*Acholeplasma laidlawii*	PG-8A	Ubiquitous	1.497	31.9	1380	Yes	CP000896.1
